# Jasmonic Acid, Not Salicyclic Acid Restricts Endophytic Root Colonization of Rice

**DOI:** 10.3389/fpls.2019.01758

**Published:** 2020-01-29

**Authors:** Xi Chen, Marta Marszałkowska, Barbara Reinhold-Hurek

**Affiliations:** Department of Microbe-Plant Interactions, Faculty of Biology and Chemistry, CBIB (Center for Biomolecular Interactions Bremen), University of Bremen, Bremen, Germany

**Keywords:** *Oryza sativa*, root endophytes, *Azoarcus olearius*, transcriptome, colonization, phytohormones, jasmonate, *Xanthomonas oryzae* pv. *oryzae*

## Abstract

Research on the interaction between the non-nodule-forming bacterial endophytes and their host plants is still in its infancy. Especially the understanding of plant control mechanisms which govern endophytic colonization is very limited. The current study sets out to determine which hormonal signaling pathway controls endophytic colonization in rice, and whether the mechanisms deviate for a pathogen. The endophyte *Azoarcus olearius* BH72—rice model was used to investigate root responses to endophytes in comparison to the recently established pathosystem of rice blight *Xanthomonas oryzae* pv. *oryzae* PXO99 (*Xoo*) in flooded roots. In the rice root transcriptome, 523 or 664 genes were found to be differentially expressed in response to *Azoarcus* or *Xoo* colonization, respectively; however, the response was drastically different, with only 6% of the differentially expressed genes (DEGs) overlapping. Overall, *Xoo* infection induced a much stronger defense reaction than *Azoarcus* colonization, with the latter leading to down-regulation of many defense related DEGs. Endophyte-induced DEGs encoded several enzymes involved in phytoalexin biosynthesis, ROS (reactive oxygen species) production, or pathogenesis-related (PR) proteins. Among putative plant markers related to signal transduction pathways modulated exclusively during *Azoarcus* colonization, none overlapped with previously published DEGs identified for another rice endophyte, *Azospirillum* sp. B510. This suggests a large variation in responses of individual genotypic combinations. Interestingly, the DEGs related to jasmonate (JA) signaling pathway were found to be consistently activated by both beneficial endophytes. In contrast, the salicylate (SA) pathway was activated only in roots infected by the pathogen. To determine the impact of SA and JA production on root colonization by the endophyte and the pathogen, rice mutants with altered hormonal responses were employed: mutant *cpm2* deficient in jasmonate synthesis, and RNA interference (RNAi) knockdown lines of *NPR1* decreased in salicylic acid-mediated defense responses (*NPR1-kd*). Only in *cpm2*, endophytic colonization of *Azoarcus* was significantly increased, while *Xoo* colonization was not affected. Surprisingly, *NPR1-kd* lines showed slightly decreased colonization by *Xoo*, contrary to published results for leaves. These outcomes suggest that JA but not SA signaling is involved in controlling the *Azoarcus* endophyte density in roots and can restrict internal root colonization, thereby shaping the beneficial root microbiome.

## Introduction

For land plants the primary site of interactions with microbes are roots; here the tissues commonly harbor the largest numbers of microbes (Reinhold-Hurek et al., 2015). The tight association with microbes often improves plants’ nutrient uptake, protects them against pathogens or even promotes their growth by the release of phytohormone-like substances (Berendsen et al., 2012). In order to profit from distinct microbial functions, plants actively establish a beneficial microbial community inside and, on the root, as well as in the rhizosphere soil e.g., by releasing metabolites and energy sources (Peiffer et al., 2013). However, the current understanding of the complex plant-microbe interactions in the rhizosphere is still in its infancy.

Among the root and rhizosphere microbes, endophytic bacteria are expected to have a particularly tight interaction with their host plant. They reside within the living tissue of a plant without substantively harming it, in a symptomless association which remained for a long time undetected. Their endophytic lifestyle is remarkable. High numbers of culturable bacterial cells in roots have been reported (up to 10^8^/g root dry weight), particularly in rice and flooded plants (Reinhold et al., 1986; Barraquio et al., 1997). In the gradient from bulk soil to the rhizosphere and endorhizosphere, the microbial community tends to have lower diversity and a higher degree of specialization toward the root interior (Reinhold-Hurek et al., 2015). Thus, endophytic bacteria are of high interest to study fundamental questions of molecular interactions but are also a mostly untapped reservoir for agro-biotechnological applications, *e.g.,* for improvement of plant growth and health (Berendsen et al., 2012; Khare et al., 2018), phytoremediation (Barac et al., 2004), or as biofertilizer.

As plant cells can commonly detect and react to bacterial molecular components (MAMPs) through plant’s innate immunity-regulated defense responses (Macho and Zipfel, 2014), it is puzzling how endophytes can overcome these responses and colonize the root interior (Reinhold-Hurek et al., 2015). Mechanisms which enable plants to select endophytic cooperative partners over pathogens are still enigmatic. Phytohormones are highly relevant for the control of plant defense responses. It has been suggested that there are three key defense-related hormones: salicylic acid (SA), jasmonate (JA), and ethylene (ET) (Grosskinsky et al., 2012). SA mainly triggers plant defense against biotrophic or hemibiotrophic, JA against necrotrophic pathogens, though there are a few exceptions (Pieterse et al., 2012; Yang et al., 2015). SA acts as one of the systemic acquired resistance inducers in leaf (Gao et al., 2015), while mostly JA and ET regulate induced systemic resistance triggered by beneficial PRGR (plant growth-promoting rhizobacteria) (Pieterse et al., 2014). Some progress has been made in elucidating how plants shape their microbiome in the model plant *Arabidopsis thaliana,* where plant factors such as salicylic acid (Lebeis et al., 2015), coumarins (Voges et al., 2019), or the plant’s phosphate status (Hiruma et al., 2016) were shown to impact the microbial communities. However, not much is known about plant factors that shape the microbiome of important crop plants, including cereals such as rice. Moreover, gene functions related to plant immune response and secondary metabolism partly differ between rice and *Arabidopsis* (De Vleesschauwer et al., 2013; Tamaoki et al., 2013; Miyamoto et al., 2016), making studies on this cereal worthwhile. Bacterial endophytes can be assessed in this model system very well, as endophytic colonization and activity are documented for rice roots beyond doubt (Hurek et al., 1994; Reinhold-Hurek and Hurek, 1998; Egener et al., 1999).

Therefore, as a model for endophyte-rice interactions, *Azoarcus olearius* BH72 was chosen, an abundant nitrogen-fixing endophyte of Kallar grass roots (Reinhold et al., 1986; Reinhold-Hurek et al., 1993b; Hurek et al., 2002), which also colonizes rice densely and fixes nitrogen in the root cortex (Hurek et al., 1994; Hurek et al., 1997; Egener et al., 1999). *Azoarcus’* root ingress is an active process to which many bacterial factors contribute, including cellulases (Reinhold-Hurek et al., 2006), type IV pili (Dörr et al., 1998) and their twitching motility (Böhm et al., 2007), type VI protein secretion (Sarkar et al., 2017), and cyclic-di-GMP-synthesizing proteins (Shidore et al., 2012). Interestingly, flagella of *A. olearius* BH72 are promoting endophytic rice root colonization, rather than acting as plant defense-inducing MAMPs (Shidore et al., 2012). With respect to the plant side, the common signaling pathway shared by nitrogen-fixing root nodule symbioses and arbuscular mycorrhizal symbioses is apparently not recruited for the establishment of the *Azoarcus* in rice (Chen et al., 2015). How rice is governing endophytic interactions, and which rice signaling cascades may facilitate or restrict endophytic colonization is still unclear. As plants can induce different panels of gene transcription during colonization by beneﬁcial or detrimental microbes (Plett and Martin, 2018), it was hypothesized that rice root reactions to endophytic and pathogenic bacteria deviate and thus allow to filter out endophyte-specific plant responses.

Thus, the main objective of this study was to disclose differences in rice root responses to beneficial endophytic and pathogenic bacteria, in order to reveal putative endophyte-specific pathways which control colonization. For this, root transcriptomic responses of rice to *A. olearius* colonization were analyzed. This required an experimental strategy to allow a direct comparison under identical experimental settings under flooded conditions typical for paddy rice. Therefore, a reference model for pathogenic plant-bacterial interactions using the highly virulent leaf blight pathogen *Xanthomonas oryzae* pv. *oryzae* PXO99 (*Xoo*) was developed in a previous study (Chen et al., 2015). Although for *X. oryzae* mostly leaf responses were studied up to now, some cell death occurs also in roots upon incubation with the pathogen (Jalmi and Sinha, 2016). It was established by us that without external artificial wounding *Xoo* is able to infect rice roots, forms colonies inside root tissues, and causes no visible damage within 14 days of infection (Chen et al., 2015). Based on the transcriptome results, two rice mutants deficient in jasmonate synthesis (*cpm2*) or exhibiting reduced salicylic acid-mediated defense responses (*NPR1-kd*) were used to study if these hormonal pathways govern colonization levels of the endophyte or the pathogen.

## Materials and Methods

### Plant and Bacterial Material

For global transcriptome experiments, rice cultivar *Oryza sativa* cv. Nipponbare (japonica type) was used (accession IRGC 136196, IRRI International Rice Research Institute, Philippines). The *cpm2* mutant was isolated from γ-ray-mutagenized M_2_ line of japonica type rice *O. sativa* cv. Nihonmasari, and the *cpm2* homozygotes with longer coleoptile under continuous light (Riemann et al., 2013) were applied for bacterial colonization experiments. *NPR1-knockdown* (*NPR1-kd*) lines #1 and #7 are RNA interference mutation lines (Sugano et al., 2010).

For microarray analysis and root colonization tests, *A. olearius* BH72 (Reinhold et al., 1986) and *X. oryzae* pv. *oryzae* PXO99 originating from Philippines (Adhikari et al., 1995) were applied. Reporter strain *A. olearius* BHGN3.1 carried a transcriptional *nifH::gusA* fusion in the chromosome (Egener et al., 1999) and was used for visualizing physiologically successful rice colonization, under which the cells can derepress nitrogenase genes and actively fix nitrogen.

### Plant Cultivation and Inoculation

Dehusked rice grains were surface sterilized, washed, and germinated on agar plates as described previously (Hurek et al., 1994) with the following modifications. Washing steps were extended to three times 1 h each. For germinating *O. sativa* japonica, rice grains were incubated in germination agar in Magenta boxes GA7 (Sigma-Aldrich, USA; 1% agar, Difco, Becton and Dickinson Company, USA) for 3 days at 30°C in the dark at ambient humidity without humidity control, followed by 2 days in light in the phytotron (see conditions below, end of paragraph). *Azoarcus* inoculation for transcriptome analysis and visualization was done as previously described in plant medium-flooded quartz sand (Egener et al., 1999), with bacterial inoculum of 2 x 10^8^ cells per plant; medium was supplemented (per liter) with 20 mg of neutralized DL-malic acid as starter carbon source, as well as potassium phosphate buffer adjusted to pH 6.8 (0.88 g KH_2_PO_4_/1.12 g K_2_HPO_4_ at pH 6.8). For timeline experiments, seedlings were instead placed on top of plastic adaptors in hydroponic jars containing 300 ml (1 h; 4 h incubation) or 450 ml (24 h, 72 h incubation) of plant medium described above. *X. oryzae* pv. *oryzae* PXO99 was grown at 28°C on agar plates containing modified Wakimoto’s medium (Karnagilla and Natural, 1973). For infection of *O. sativa* with the pathogen *Xoo* PXO99, roots of seedlings were dipped for 5 min into a bacterial suspension of 5 x 10^9^ cells/ml. Afterwards infected seedlings as well as non-infected seedlings (control) were grown gnotobiotically as described above. Plants were incubated in the phytotron at 30°C, 60% humidity, and 14/10-h light-dark cycle (approximately 170 µmol photons/m^−2^ x s^−1^).

### Assessment of Colonization

Endophytic bacterial colonization (inside the roots) was quantified as described previously (Böhm et al., 2007); briefly, 14 days after inoculation, roots were treated by ultrasonication to remove surface bacteria, homogenized, and the number of colony forming units (cfu) per milligram of root fresh weight was estimated for both bacteria, *Azoarcus* (according to Böhm et al., 2007) and PXO99. Pathogen PXO99 was counted on Wakimoto’s medium agar plates (Karnagilla and Natural, 1973; Chen et al., 2015).

For histochemical detection of ß-glucuronidase (GUS) activity, roots were harvested 13 days post-inoculation and stained for up to 6 h as previously described (Egener et al., 1999). Roots from three independent experiments were inspected.

### Ribonucleic Acid Extraction and Transcript Analysis by Real-Time Polymerase Chain Reaction

Plants were harvested 14 days post-inoculation, and roots were frozen in liquid nitrogen prior to RNA extraction from pools of plants. RNA applied for microarray experiments was extracted by using a hexadecyl trimethyl-ammonium bromide (CTAB)-based method: 0.5 g rice roots homogenized in liquid nitrogen were suspended in 18 ml of extraction buffer (2% hexadecyl trimethyl-ammonium bromide (CTAB), 2% polyvinylpyrrolidone, 100 mM Tris-HCl pH 8.0, 25 mM EDTA, 2 M NaCl, 0.5 g/L spermidine, and 2% β-mercaptoethanol, incubated at 65°C for 5 minutes; 18 ml of chloroform was added and mixed with the suspension; after centrifugation at 10,000 x g for 5 min, the supernatant was treated with chloroform again; lithium chloride (LiCl) was added to the final supernatant to a final concentration of 2 M and kept overnight at 4°C for RNA precipitation; RNA was pelleted at 10,000 x g for 30 min at 4°C and dissolved in RNase-free water.

RNA applied for timeline experiments was extracted with the RNAeasy Plus Mini Kit after homogenizing the root samples in liquid nitrogen. Samples contaminated with genomic DNA were subjected to DNase I treatment (Sigma-Aldrich, St. Louis, Missouri, USA).

For quantitative real time (RT)-PCR analysis, accession numbers of the respective rice genes and primer sequences are given in **Table S1**. The reverse transcription step was performed using Thermo Scientific RevertAid Premium Transcriptase (Thermo Fisher Scientific, Waltham, MA, USA) according to the manufacturer’s instructions with and 1 μg RNA applied for 20 μl reaction volume. Real-time PCR was carried out either with Bio-Rad SsoAdvanced SYBR Green Supermix, with 300 nM of each primer and 2 μl of complementary DNA (cDNA) added. Real-time PCR reactions were performed either using a CFX96 Touch Real-Time Detection System (Bio-Rad, Munich, Germany) at 30 s of initial denaturation at 95°C, followed by 40 cycles of denaturation for 10 s at 95°C, annealing for 30 s, and extension for 30 s at 72°C. At the end of the amplification, a melting curve was recorded between 55 to 95°C in steps of 1°C, to ensure that the signal corresponded to a single PCR product. As the efficiency of each real-time PCR amplification was close to 100%, relative gene expression was calculated with 2-ΔΔ*CT* method (Livak and Schmittgen, 2001).

### Microarray Hybridization and Data Collection

For microarray hybridization, rice RNA samples were subjected to quality control using an Agilent Bioanalyzer 2100. Only those showing no degradation and clear 28S ribosomal RNA (rRNA) and 18S rRNA peaks were used. A two-color microarray-based analysis with Low Input Quick Amp Labeling kit and 4×44 k 60-mer microarrays (Agilent; Böblingen, Germany) was carried out according to the company’s instructions. The data extractions were performed with Feature Extraction Software version 9.5 (Agilent; Böblingen, Germany) and GeneSpring software (Agilent; Böblingen, Germany). For each experiment three biological replicates were performed, and for hybridization one dye-swap and a technical replicate were included. Genes showing equal or larger than 1.5-fold up- or down-regulation in all three experiments were regarded as differently regulated. Data were deposited at GEO (Gene Expression Omnibus) (GSE136706 and GSE136707).

## Results and Discussion

### Highly Divergent Global Transcriptomic Response of Rice Roots Toward Bacterial Endophyte or Pathogen

The current study aimed to compare plant responses to beneficial and pathogenic bacteria with similar surface characteristics or microbe-associated molecular patterns, such as an outer membrane with lipopolysaccharides which is typical for Gram-negative bacteria. As counterpart for *A. olearius*, a Gram-negative endophyte of rice roots, the Gram-negative leaf pathogen *X. oryzae* pv. *oryzae* strain PXO99 (*Xoo*) was chosen. It was possible to utilize this strain for root responses, because it was previously demonstrated (Chen et al., 2015) that it can also colonize rice roots in high numbers. Root transcriptomic responses were analyzed by two-color 4×44 k rice microarrays (Agilent; Böblingen, Germany). They were examined 2 weeks post-inoculation with bacteria under gnotobiotic conditions when nitrogen-fixing endophytic colonization is well detectable (Egener et al., 1999; Chen et al., 2015). This allowed simultaneous analysis of both, local early and late responses of roots to new local infections and fully established endophytes. As differentially expressed genes (DEGs), genes which showed at least 1.5 fold difference in all three replicates were considered.

**Figure 1:**
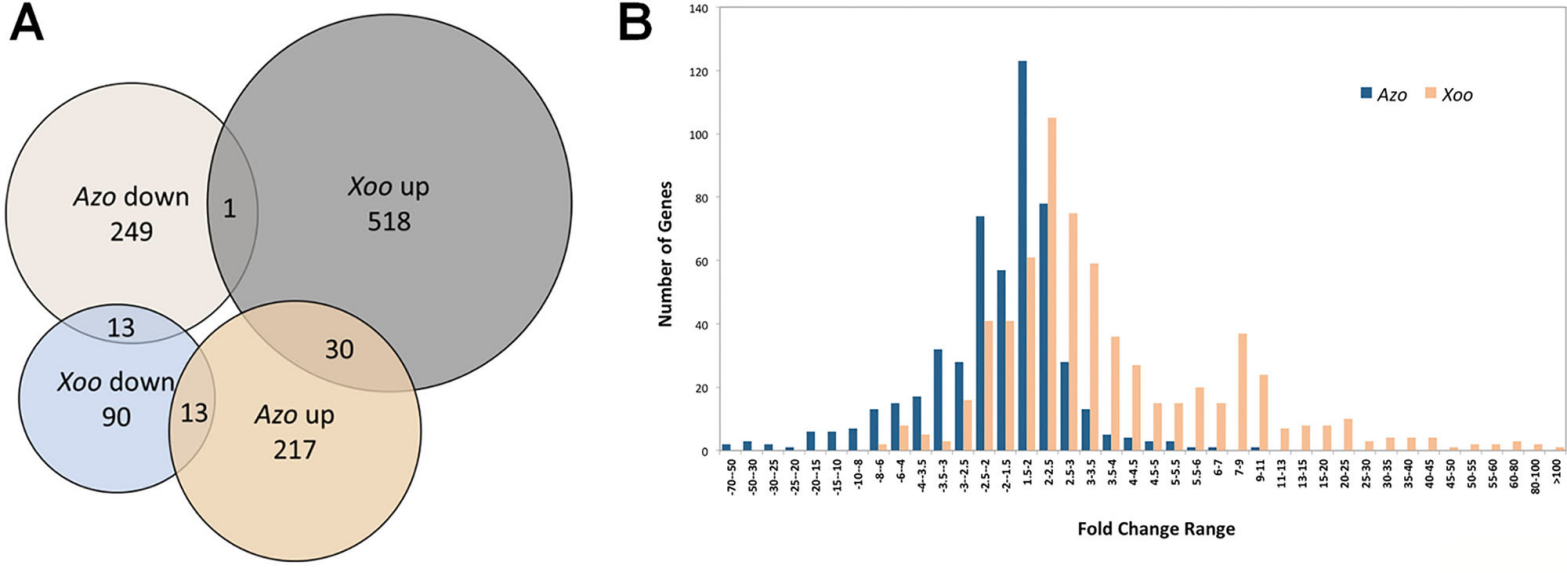
Overview of rice genes differentially regulated in roots in response to colonization by *Azoarcus olearius* BH72 (*Azo*) and *Xanthomonas oryza* pv. *oryzae* PXO99 (*Xoo*). **(A)** Venn gram of genes differently regulated in response to *Azoarcus* and *Xoo.* In total 1,227 genes were differentially regulated. Differentially expressed genes (DEGs) that were up-regulated are referred to as “up,” down-regulated as “down.” **(B)** Gene number distribution at different fold change ranges. Orange color represents number of differently regulated genes in response to *Azoarcus,* blue color in response to *Xoo*.

In total 523 genes were found to be differently regulated in response to the endophyte *A. olearius* BH72 compared to non-infected, sterile seedlings, with 260 up-regulated and 263 down-regulated genes. For the pathogen *X. oryzae* strain PXO99 (*Xoo*), the number of modulated genes (664) was almost equal to *Azoarcus*, albeit five times more genes were up- than down-regulated (549 *versus* 116) (**Figure 1A**). Six DEGs each, for pathogen and endophyte, were chosen for real-time polymerase chain reaction (PCR) to validate transcriptional changes (**Table 1**).

**Table 1:** Quantitative real-time (RT)-PCR analysis of transcriptional regulation of selected genes.

	RT-qPCR*	Microarray*	Annotation
***Azoarcus***			
**Os03g0667100**	2.3 ± 0.9	1.6 ± 0.2	OsNPR3
**Os06g0317200**	249 ± 310	−79 ± 70	Similar to glycine-rich cell wall structural protein
**Os06g0592500**	−7 ± 3	−9 ± 4	Similar to Ethylene-responsive transcriptional coactivator
**Os06g0695300**	−6 ± 2	−6 ± 4	prx92; class III peroxidase 92
**Os09g0483300**	−4,4 ± 2.9	−2.2 ± 0.8	Calcium-binding EF hand family protein
**Os11g0242800**	−2,2 ± 0.8	−1,8 ± 0.1	light-harvesting protein ASCAB9-A, PSII CP26, PSII Lhcb5
***Xoo***			
**Os02g0587800**	387 ± 489	78 ± 80	Virulence factor, pectin lyase fold family protein
**Os05g0368000**	3.1 ± 1.7	2.2 ± 0.5	RH1; NRR repressor homologue 1
**Os06g0592500**	12 ± 5	9 ± 5	Similar to ethylene-responsive transcriptional coactivator
**Os06g0695300**	50 ± 34	26 ± 12	prx92; class III peroxidase 92
**Os08g0535200**	3,704 ± 1,908	67 ± 31	Xa13, Os8N3, 8N3, *OsSWEET11*, SWEET11
**Os09g0483300**	24 ± 19	11 ± 9	EF hand domain containing protein

*Average fold change and standard deviation from the three biological replicates, compared for quantitative RT-PCR and microarray analysis of rice roots upon colonization with *Azoarcus* and Xoo.

Differentially regulated genes were highly divergent between endophyte and pathogen. With 6.3% of the pathogen-modulated genes, only very few DEGs overlapped in both interactions: 30 genes (2.3 %) were up-regulated and 13 genes (1%) were down-regulated. Several genes (14) were affected in the opposite way (**Figure 1A**, **Table S2**). Generally, the up-regulated genes in response to *Xoo* infection showed a much higher induction ratio than in response to *Azoarcus*. In contrast, down-regulated genes were generally more strongly repressed by the endophyte (**Figure 1B**). The root responses were also distinct with respect to functional categories of differentially regulated genes deduced by Kyoto Encyclopedia of Genes and Genomes (KEGG) (**Figure S1**). In the case of *Xoo* infection, a high cumulative fold change was found for gene induction in almost all functional categories. Contrastingly, the majority of categories showed a high cumulative fold change for genes repressed by *Azoarcus*. This included genes in functional categories related to the cell wall, stress, or major CHO metabolism, which were largely down-regulated in the presence of endophytic bacteria. This demonstrates that rice responds with different patterns of gene regulation to colonization by beneﬁcial or detrimental bacteria.

### Moderate Plant Defense Signaling Toward the Endophyte in Comparison to the Pathogen

**Figure 2:**
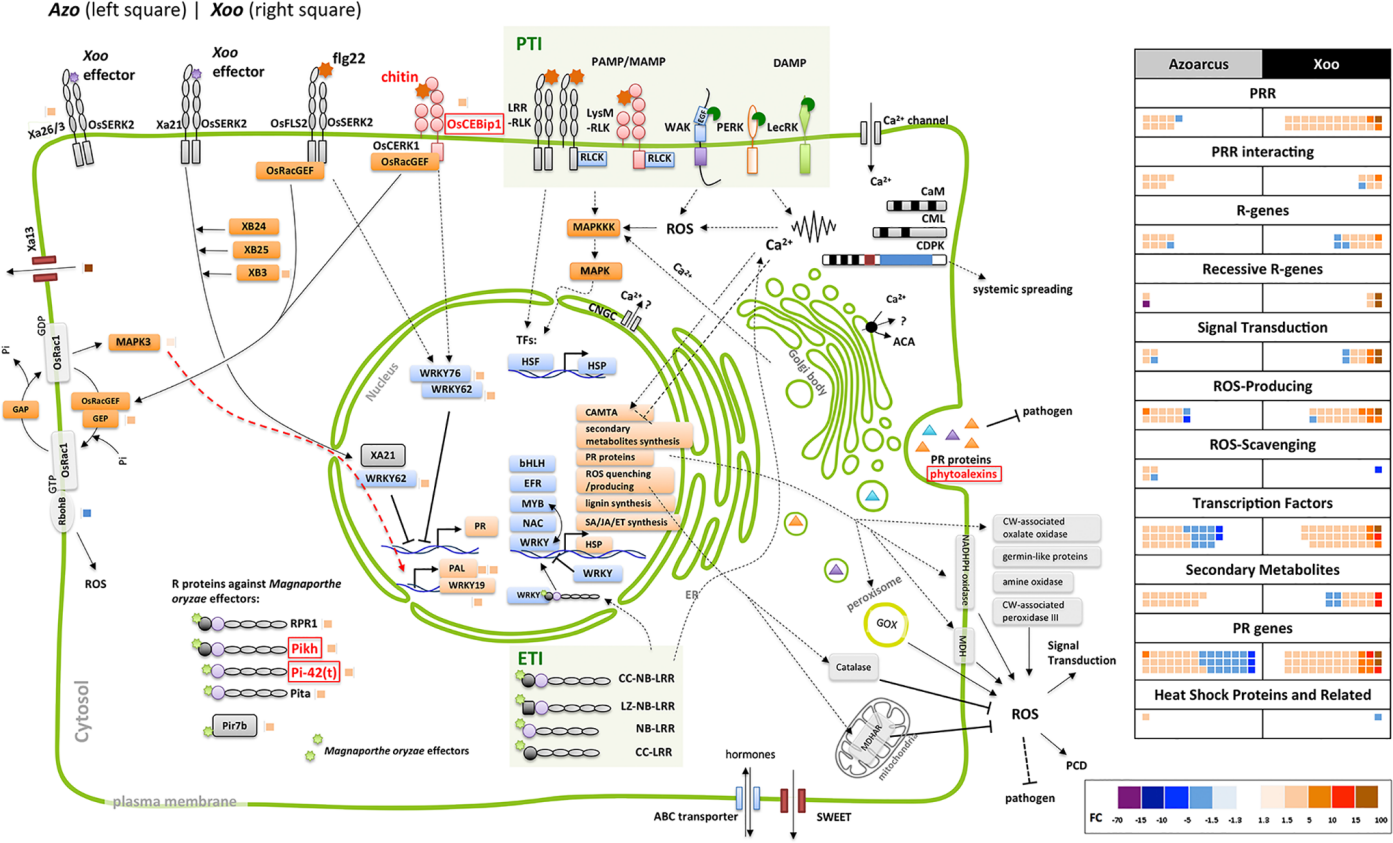
Elements of plant defense reactions modulated in *Azoarcus*- and *Xoo*-rice interactions in roots. Sketch of putative rice cellular defense network (left side). Dashed line: direct interaction not verified or indirect interaction; continuous line: direct interaction; arrow: induction, blunt end: inhibition. Small blocks beside rice protein names: Fold change of the differentially expressed gene (DEG) (right side of the vertical line: modulated by *Xoo*, left side: by *Azoarcus*) indicated by its color according to the color scale below. Genes or pathways up-regulated by *Azoarcus olearius* labeled in red. Right side, summary of defense-related DEGs modulated by *Azo* or *Xoo*, respectively; each colored block represents a modulated gene, its color indicating the fold change to the color scale below. ABA, abscisic acid; ABC, ATP-binding cassette; ACA, auto-inhibited *calcium* ATPase; bHLH, basic helix-loop-helix protein; BR, brassinosteroid; CaM, calmodulin; CAMTA, Ca^2+^/CaM-binding transcription factors; CC, coiled coil; CDPK, Ca^2+^-dependent protein kinases; CEBip, chitin elicitor-binding protein; CERK, chitin elicitor receptor kinase; CK, cytokinin; CML, calmodulin-like; CNGC, cyclic nucleotide-gated channels; CW, cell wall; DAMP, damage-associated molecular pattern; ER, endoplasmic reticulum; ERF, ethylene response transcription factor; ET, ethylene; flg, flagellin; FLS, flagellin-sensing; GA, gibberellic acid; GAP, GTPase-activating protein; GEF, guanine nucleotide exchange factor; GEP, GDP/GTP exchange protein; GOX, glycolate oxidases; HSF, heat stress transcription factor; HSP, heat shock protein; IAA, indole-3-acetic acid; JA, jasmonate; LecRK, lectin receptor kinase; LRR, leucine rich repeat; LysM, lysine motif domain; LZ, leucine zipper nucleotide-binding site; MAPK, mitogen-activated protein kinase; MDHAR, monodehydroascorbate reductase; *M.o*., *Magnaporthe oryzae* MYB, myeloblastosis transcription factor family; NAC, no apical meristem (NAM); ATAF, *Arabidopsis* transcription activation factor; CUC and cup-shaped cotyledon transcription factor family; NBS, nucleotide-binding site; Os, *Oryza sativa;* PAL, phenylalanine ammonia lyase; PAMP/MAMP, pathogen/microbe-associated molecular pattern; PCD, programmed cell death; PERK, proline extension-like receptor kinase1; Pi, *Pyricularia oryzae* resistance; PR genes, pathogenesis-related genes; PRR, pattern recognition receptors; PTI, PRR-triggered immunity; R, resistance; Rac, Ras-related C3 botulinum toxin substrate; RbohB, respiratory burst oxidase homolog B; RLCK, receptor-like cytoplasmic kinases; RLK, receptor like kinases; ROS, reactive oxygen species; RPR, rice probenazol responsible; SA, salicylic acid; SERK, somatic embryogenesis receptor kinase; SWEET, sugars will eventually be exported transporters; syn, synthesis; TF, transcription factors; WAK, cell wall-associated kinase; Xa, *Xanthomonas campestri*s pv. *oryzae* resistance; XB, XA21 binding proteins.

Plant defense reactions were induced both by the endophyte and the pathogen colonization. However, the defense responses were weaker toward the endophyte, with respect to the number and type of DEGs as well as in the degree or trend of modulation (**Table S3**, **Figure 2**).

The first set of analyses was aimed at comparing the expression patters of genes encoding pattern recognition receptors (PRRs). These receptors mediate the first line of plant defense response. PRRs recognize microbe- or pathogen-associated molecular patterns from invading microbe (MAMPs/DAMPs) or damage-associated molecular patterns (DAMPs) released from plants upon damage by invading microbes (Macho and Zipfel, 2014), which induces PAMP-triggered immunity (PTI). The largest group of detected PRR-related DEGs encoded leucine-rich-repeat receptor-like kinases (LRR-RLKs), typically involved in the perception of classical MAMPs/PAMPs like e.g., bacterial flagellin, elongation factor Tu (EF-Tu), or endogenous Pep peptides (Ma et al., 2012; Macho and Zipfel, 2014). Interestingly, endophytic colonization led to moderate modulation of expression of only five DEGs encoding LRR-RLKs, while pathogen infection stipulated strong upregulation of 11 DEGs encoding LRR-RLKs (**Table S3**). Expression of only one gene (*Os04g0227000)* was upregulated by both, endophyte and pathogen.

Another group of detected DEGs encoding PRRs included lectin receptor kinases (LecRKs) known for their role in binding various carbohydrates, WAK proteins found to bind glycine-rich proteins (GRPs), pectin or oligogalacturonides (OGs) released from cell walls (Brutus et al., 2010; Delteil et al., 2016), and proline extension-like receptor kinase 1 (PERK1) for MAMP/PAMP and/or DAMP detection (Silva and Goring, 2002). Similarly, genes encoding lysine motif domain (LysM) domain-containing receptor kinases, out of which some detect peptidoglycan (PGN) or chitin like OsCERK1, OsLYP4, and OsLYP6 (Ao et al., 2014), were differently modulated. DEGs encoding all abovementioned groups of PRRs were strongly induced by pathogen infection, while only one member of each group was weakly modulated by *Azoarcus* colonization (**Table S3**). Therefore, it can be speculated that endophytic colonization may be less damaging to rice roots in comparison to pathogen infection (less DAMPs), or DAMP or PAMP signaling in mutualistic interactions might be masked or blocked as suggested previously (Plett and Martin, 2018).

*Azoarcus* perception might involve chitin perception receptor OsCEBiP1 (Akamatsu et al., 2013) and downstream Mitogen-Activated Protein Kinase 3 (*MAPK3*), as genes encoding both of these receptors were upregulated during *Azoarcus* colonization (**Figure 2**). Interestingly, in rice suspension culture chitin is able to induce production of jasmonic acid and phytoalexin (Kaku and Shibuya, 2016), which aligns with the transcriptional activation of genes related to JA and phytoalexin biosynthesis observed during rice colonization by *Azoarcus*. The LYP4 and LYP6 participating in peptidoglycan perception (Liu et al., 2012) were, however, not induced.

Pathogen infection induced more DEGs encoding receptor-like cytoplasmic kinases (RLCK, 5 DEGs) than endophytes (only one DEG) (**Table S3**). The signal transduction during PTI typically requires PRRs to phosphorylate RLCKs which, in turn, leads to mitogen-activated protein kinase (MAPK)-dependent or -independent ROS burst and defense gene expression (**Figure 2**) (Macho and Zipfel, 2014).

Interestingly, it has been demonstrated that MAPK signaling can be negatively regulated by protein phosphatase 2C (PP2C), like in case of kinase-associated protein phosphatase (KAPP) interacting with FLS2 and reducing the flg22-induced immune responses (Gómez-Gómez et al., 2001; Park et al., 2008). A weak but stable upregulation of expression of four genes encoding PP2C homologues was detected upon *Azoarcus* colonization, and downregulation of another PP2C homologue in response to *Xoo* infection (**Table S3**). This specific modulation may be linked to the observed differences in strength of PTI response to a pathogen and an endophyte.

The second layer of the plant immune system is effector-triggered immunity (ETI). As a result of coevolution, plant pathogens produce virulence factors called effectors to modulate the PTI. Correspondingly, plants also evolved a family of the polymorphic intracellular nucleotide-binding site and leucine-rich repeat domain-containing proteins (NBS-LRRs or NLRs), known as resistance proteins (R proteins), to perceive pathogen effectors and induce ETI (Cui et al., 2015). Accordingly, the pathogen induced more and different R genes (eight DEGs) in comparison to the endophyte (three DEGs) (**Table S3**).

### Divergent Signal Transduction in Endophyte- and Pathogen Induced Responses

As Ca^2+^ concentration change, activation of MAPKs and transcription factors are among earliest components of signaling pathways during plant defense responses (Meng and Zhang, 2013); the expression patterns of genes related to these processes between rice roots colonized by *Xoo* and *Azoarcus* were compared.

It was observed that *Xoo* infection led to a strong induction of 10 DEGs associated with calcium signaling, including two genes encoding EF-Hand type domain-containing proteins (Os09g0483500, Os09g0483100), exhibiting high FC of 20.1 and 55.8, respectively (**Table S3**). They are also strongly upregulated in rice overexpressing transcription factor *OsERF71* which is linked to drought resistance, but not to biotic stress (Lee et al., 2016). Only four genes which belong to this category were moderately modulated by *Azoarcus* (**Table S3**). No overlapping DEGs were found for this category, which indicates differences in calcium signaling utilization in transcriptomic response to the endophyte and the pathogen.

Also, the expression patterns of plant transcription factor expression (TF) were hardly overlapping between rice roots colonized by the endophyte and plants colonized by the pathogen. Overall, more DEGs encoding for to AP2/ERF, bHLH, bZIP/TGA, MYB, and WRKY family were up-regulated by *Xoo* infection in each family, generally also with a higher FC. In contrast, genes belonging to these families were mostly down-regulated during *Azoarcus* colonization, some exhibiting high FC value (**Table S3**). One notable exception includes the DEGs encoding the NAC-TF-family, which were almost exclusively upregulated by *Azoarcus*. NAC TFs are a large group of genes, comprising 151 homologues in rice, playing various roles in rice biotic and abiotic responses. There is, however, very limited data from previous studies regarding *Azoarcus*-responsive *NACs* genes, with only two characterized DEGs including cold-induced *OsONAC059* and salinity-stress induced *OsONAC103* (Fang et al., 2008).

Genes encoding MAPKs which met the criteria for DEGs were not detected, however a gene encoding OsMAPK3 was exhibiting stable expression induction of 1.3-fold only in *Azoarcus*-colonized plants among all technical and biological replicates (left side of **Figure 2** and **Table S4**). It remains to be investigated whether OsMAPK3 gene product, involved in resistance to abiotic stress like chilling (Zhang et al., 2017), is also involved in signal transduction in roots subjected to endophyte colonization.

Taken together, the analysis of signaling-related DEGs shows a strong difference in perception of pathogen and endophyte by the rice plant, with the latter inducing weaker responses, in many cases leading to transcriptional repression of signaling genes.

### Most Downstream Defense Reactions Repressed in the Endophytic Interaction

PTI signaling leads to various cellular responses and physiological changes in plants including ROS production, induced cell wall fortification, biosynthesis of antimicrobial secondary metabolites, and upregulation of specific pathogen-related genes (Grosskinsky et al., 2012). Pronounced differences in expression patterns of genes governing these processes were observed between roots colonized by *Xoo* and *Azoarcus*. Many ROS-related DEGs encoding type III peroxidases, oxalate oxidases, germin-like proteins, and amine oxidases which were induced by the endophyte or the pathogen were detected (**Table S3**). Interestingly, endophyte induced less (12) DEGs with moderate FC (1.5–5.6x) than pathogen, which has induced more DEGs (18) with higher FC (2.1–14x). For the type III peroxidase gene *OsPrx92* a very pronounced difference in expression was detected, as this gene was downregulated (−5.6x) by the endophyte and upregulated (+14.0x) by the pathogen. With respect to ROS scavenging, only one gene was upregulated by the endophyte (*OsGRX9*), while *Os08g0470700* was strongly downregulated by the pathogen. ROS play a vital role in plant immunity as they prime plants against pathogens not only *via* localized oxidative bursts but also as a sustained ROS signaling system (Camejo et al., 2016). It can be speculated that weak ROS-related transcriptomic response in case of *Azoarcus*-colonized roots could further decrease defense-related systemic signaling, leading to lack of symptoms of pathogenicity in these roots.

To build up direct barrier against bacterial penetration, plants induce processes such as callose deposition and lignin synthesis. *Azoarcus* and *Xoo* both induced a group of genes related to lignin biosynthesis, though in *Xoo* infection to a much higher expression level (**Figure 2**, **Tables S3** and **S10**). Several genes related to glycosyl hydrolases were down-regulated by *Azoarcus* only (**Table S10**), suggesting that by suppression of genes related to degradation, plant cell walls are strengthened against the endophyte.

Pathogenesis-related (PR) proteins are divergent set of proteins that are induced as a result of signaling upon pathogen infection. At least 17 groups of PR proteins are recognized in plants, and 13 groups of them were found differently regulated in *Azoarcus* and *Xoo*-rice interactions. Similarly, expression of this group of genes was more strongly upregulated by *Xoo* than by *Azoarcus* (**Figure 2**, **Table S3**). During *Azoarcus* colonization, genes coding for PR 1, 2, 3, 9, 10, 15, and 16 were mainly up-regulated. By *Xoo* infection, also genes coding for PR alpha, 1, 2, 3, 5, 8, 9, 10, 12, 13, 14, 15, and 16 were up-regulated, some of them strongly, like *Os06g0695300* (encoding PR9, 14-fold), *Os03g0700100* (encoding PR13, 44.1-fold), and *Os07g0215500* (encoding PR14, 58.3-fold). Genes encoding PR 5 and 8 were only induced in case of *Xoo* infection. In contrast, genes encoding PR6, 13, and PR14 were mainly down-regulated by the endophyte (**Table S3**). Among 77 PR-encoding DEGs detected in *Azoarcus*-treated and *Xoo*-treated roots, only 5 DEGs exhibited similar expression pattern upon colonization by both bacteria, highlighting the strong difference between transcriptomic response to a pathogen and to an endophyte.

Defensin-like peptides called nodule-specific cysteine-rich peptides (NCR) can possess antimicrobial functions but also control rhizobial differentiation to increase efficiency of nitrogen fixation in root nodules of legumes (Maroti et al., 2015). Typical motifs for NCRs were found for *Os04g0381500* (**Figure S2**), which was 1.5 fold upregulated by the endophyte but not modulated by *Xoo* (**Table S3**) and could have a potential role in *Azoarcus*-rice mutualism.

Another group of DEGs exhibiting strong difference in expression between the roots colonized by *Xoo* and *Azoarcus,* was a group of heat-shock protein-encoding genes. They are molecular chaperones, typically involved in heat resistance by disaggregating or degrading non-functional proteins and degrading irreversibly damaged polypeptides. They are also playing a role in resistance during HR (for example: OsHSP70, OsHSP40), or by functioning in HR, or interact with cytosolic R proteins (HSP90) (Guo et al., 2016). Interestingly, *Azoarcus* colonization led to down-regulation of two HSP-encoding genes (OsHSP70, OsHSP40), while *Xoo* strongly induced expression of *OsHSP100* and *OsHSP90*, three small *OsHSPs,* and reduced expression of another *OsHSP90* (**Figure 2**, **Table S3**).

**Figure 3:**
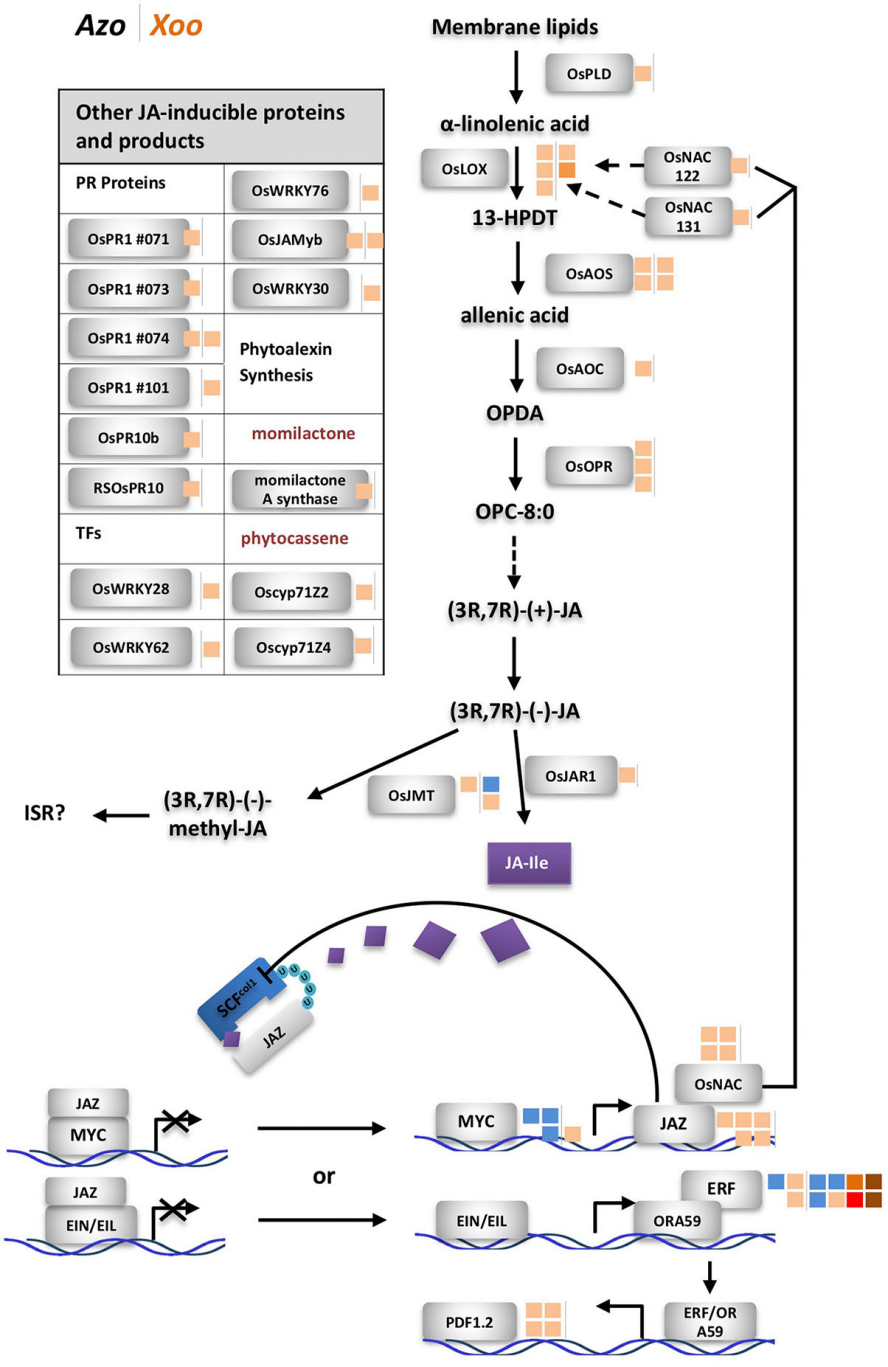
Differentially expressed genes related to jasmonate (JA) biosynthesis and downstream reactions. Next to rice protein names small blocks representing fold change of the differentially expressed gene (DEG) (right side of the vertical line, modulated by *Xoo*; left side, by *Azoarcus*) according to the color scale below. Dashed line: steps omitted, continuous line: direct reaction, arrow: reaction or induction, blunt end: inhibition. Left side, modulated DEGs not shown in the sketch; colored blocks indicating the fold change according to the color scale below. FC and annotation of DEGs from **Table S5**. AOC, allene oxide cyclase; AOS, allene oxide synthase; cyp, cytochrome P450; EIL, ethylene insensitive-3 (EIN3)-like; EIN, ethylene insensitive; ERF, ethylene response factor; FC, fold change; 13-HPDT, 13S-hydroperoxy-(9Z;11E;15)-octadecatrienoic acid; ISR, induced systemic resistance; JA, jasmonate; JA-Ile, jasmonoyl-isoleucine; JAMyb, JA-regulated myb transcription factor; JAR, jasmonate resistance; JAZ, jasmonate ZIM domain-containing; JMT, jasmonic acid methyl transferase; LOX, lipoxygenase; NAC, no apical meristem (NAM); OPC-8,0, 3-oxo-2-(20(Z)-pentenyl)-cyclopentane-1-octanoic acid; OPDA, oxophytodienoic acid; OPR, OPDA reductase; ORA, octadecanoid-responsive APETALA2 (AP2)/ERF; Os, *Oryza sativa;* PLD, phospholipase D; PR, pathogenesis related; RSOsPR, root-specific *Oryza sativa* PR; SCF, Skp1, Cullin, and F-box-containing complex; U, ubiquitinylated protein.

Surprisingly, a group of defense-related genes encoding enzymes involved in phytoalexin biosynthesis was detected that was exclusively induced by the endophyte colonization. Phytoalexins are antimicrobial secondary metabolites which accumulate at sites of pathogen infection in plants (Yamane, 2013; Miyamoto et al., 2016). *Azoarcus* induced expression of genes involved in biosynthesis of phytoalexins such as momilactones, oryzalexins S, and phytocassanes (five DEGs). Contrastingly, *Xoo* colonization weakly modulated expression of two DEGs encoding ent-isokaurene C2-hydroxylase-like protein involved in phytoanticipin oryzalide A biosynthesis (**Figure 3**, **Table S3**). As previous studies reported enhanced expression of phytoalexin-biosynthesis enzymes upon *Xoo* infection in rice leaves (data retrieved from RiceXPro database), lack of strong induction of genes encoding these enzymes might be linked to differences in tissue-specific expression patterns.

Also, alkaloids and anthocyanidins were identified by the Plant Metabolic Network tool as potential antimicrobial molecules (Chae et al., 2012). We have detected DEGs encoding strictosidine- and anthodyanidin-related enzymes, which were exclusively induced by *Azoarcus* colonization, and antioxidant-related DEGs which were only modulated by *Xoo* colonization (**Figure 2**, **Table S3**). Thus, also downstream defense reactions to the endophyte were weak, except for genes related to synthesis of secondary metabolites.

### Dominating Role of Jasmonate-Related Defense Reactions Toward Endophytic Colonization

Jasmonate-related genes comprised the major DEGs affected by the endophyte, as summarized in **Figure 3**, from data in **Table S5**. Multiple DEGs encoding JA-biosynthesis genes were detected: phospholipase D (*OsPLD*), lipoxygenase (*OsLOX*), allene oxide synthase (*OsAOS*), allene oxide cyclase (*OsAOC*), oxophytodienoic acid (*OPDA*) reductase (*OsOPR*), and jasmonate resistance 1 (*OsJAR1*). Additionally, two genes *OsNAC122* and 131 which positively regulate the expression of *OsLOX*, were up-regulated in roots colonized by *Azoarcus*. Also, upregulation of expression of genes encoding several JA-inducible proteins including PR-genes and genes encoding transcription factors was observed. Expression of some of them is uniquely governed by JA, such as *OsPR1#71*, *#73,* and *#74* and *OsJAmyb* genes (Lee et al., 2001; Mitsuhara et al., 2008). Other hormonal pathways appeared unaffected or weakly affected by the endophyte (ethylene, ET; gibberellic acid, GA; cytokinins, CK; brassinosteroids, BR), or slightly down-regulated (abscisic acid ABA, auxin indole-3-acetic acid (IAA) known for competitive or antagonistic action to JA in plants (Wang and Irving; 2011) (**Figure S3**, **Table S5**). Therefore, it can be concluded that in *Azoarcus*-rice interactions, jasmonate appeared to play a dominating role in governing the defense response.

Interestingly, only few JA-related genes were induced by *Xoo* including two DEGs encoding allene oxide synthase (*OsAOS*) and two DEGs encoding lipoxygenase (*OsLOX*). Instead, *Xoo* infection had a strong impact on expression of salicylic acid-related genes, of which only few were moderately modulated by *Azoarcus* colonization (**Figure S4**, **Table S5**). Moreover, genes involved in BR and CK biosynthesis and signaling and GA pathways were up-regulated during *Xoo* colonization. This further underlines the hypothesis that pathogen and endophyte rewire hormonal responses differently.

The current observations on hormonal responses in response to the endophyte were validated by quantifying messenger RNA (mRNA) levels of genes participating in JA, SA, ET, and ABA hormone biosynthesis and corresponding downstream reactions by quantitative RT-PCR in a timeline of colonization (1 h, 24 h, 72 h, and 7 days post-inoculation). For each hormone, one gene encoding a protein involved in biosynthesis and one located downstream were chosen for the test: *isochorismate synthase 1* (*OsICS1*) (Nahar et al., 2012; Choi et al., 2015) and *OsWRKY45* ((Nahar et al., 2012) for SA, *OsJAR1* (Svyatyna and Riemann, 2012; Lyons et al., 2013) and *OsJAmyb* (Lee et al., 2001) for JA, *acyl-CoA synthetase 2* (*OsACS2*) (Helliwell et al., 2013) and *SHR5* (Ma et al., 2013) for ET, and *9-cis-epoxycarotenoid dioxygenase 3 (OsNCED3*) (Nahar et al., 2012) and *OsMAPK5* (De Vleesschauwer et al., 2014) for ABA. Especially *OsICS1* and *OsACS2* were induced by pathogen infection as reported by previous studies (Nahar et al., 2012; Choi et al., 2015). During the infection process, only *OsJAR1*, representative for the JA-pathway [turning JA into active form jasmonoyl-isoleucine (JA-Ile)], responded significantly in three independent experiments: it was immediately up-regulated 1 h post-infection; though at 72 h, induction had seized, it rose again at later stage, 7 days post-infection (**Figure S5**). Expression of marker genes for other hormonal pathways did not respond consistently, except for *OsNCED3* which was induced 1 h post-inoculation only (**Figure S5**).

Induction of JA-related defense responses appears to be a more general feature of bacterial endophytes. The JA pathway is induced in interactions between rice and many endophytic PGPR, though not all of them (Nadarajah, 2016). In our previous study on the same *Japonica* rice cultivar, RT-PCR analysis showed induction of marker genes *OsJAR1* and *OsJAmyb* by *A. olearius* (Chen et al., 2015). In *Indica* varieties IR36 and IR42, JA-inducible proteins were overexpressed in proteome studies (Miché et al., 2006). Also, diazotrophic endophytes *Azospirillum* B510 (Drogue et al., 2014) and *Gluconacetobacter diazotrophicus* (Alquéres et al., 2013) induced JA-marker genes. In contrast, in *Arabidopsis* roots, JA-signaling was downregulated by *Azospirillum brasilense* 245 (Spaepen et al., 2014). How mutualistic microbes modulate defense responses—through effector proteins, small interfering RNAs (siRNAs) or other molecules, is still not clear (Plett and Martin, 2018).

### Jasmonate-Related but not Salicylate-Related Pathways Control Endophytic Root Colonization of *Azoarcus* in Contrast to *Xoo*

**Figure 4:**
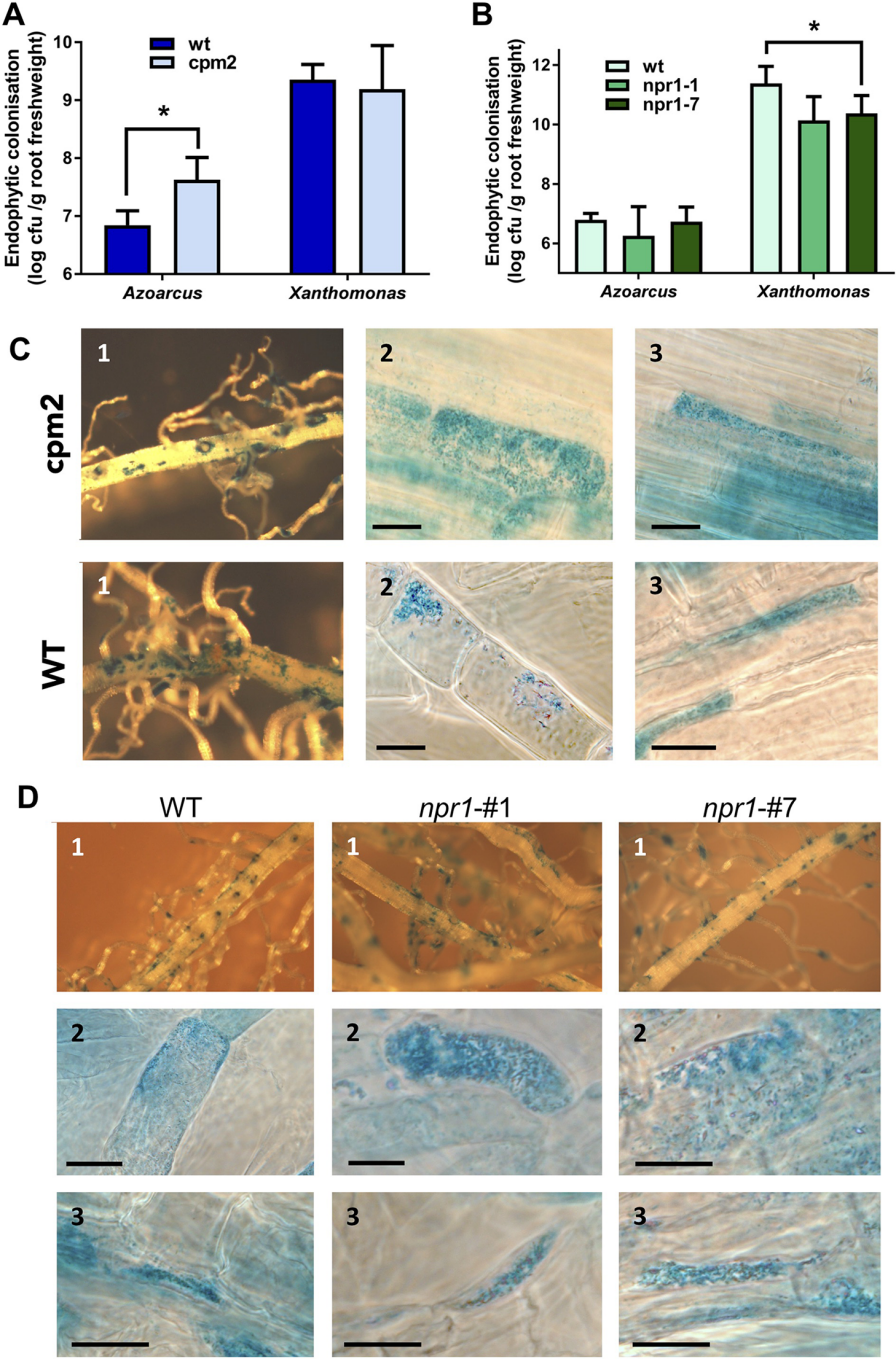
Root colonization of *Azoarcus* and *Xoo* in wild type and mutant rice altered in jasomonate (JA) and salicylate (SA) pathways. Jasmonate-deficient mutant *cpm2* and parent *Oryza sativa* cv. Nihonmasare **(A**–**C)**, or OsNPR1 knockdown mutant lines *npr1#1* and *npr1#7* and wild type cv. Nipponbare **(A, B, D)**, respectively, were inoculated and harvested 14 days post-inoculation. **(A)** Quantitative assessment of endophytic root colonization of mutant and wild-type plants by *Azoarcus olearius* BH72, or by **(B)** *Xanthomonas oryzae* pv. PXO99. Bacteria colonizing the root interior were re-isolated after surface sterilization, and colony-forming units per gram root fresh weight were counted. Data from three independent biological experiments with 7–10 plants each (mean + SD). Significance according to two-tailed paired t-test (*P* < 0.05) is indicated by star*. Differences of cell counts in **(A)** were also significant within each of the three independent experiments. **(C, D)** Histochemical ß-glucuronidase (GUS) staining of roots inoculated with the *nifH::gusA* reporter strain *A. olearius* BHGN3.1. Examples from inspection of roots from three independent experiments. **(C)** Wild type rice (WT) (Nihonmasari) and *cpm2* mutant; **(D)**, wild type rice WT (Nipponbare), and mutant lines *npr1#1* and *npr1#7*; 1, overview; 2, intracellular colonization; 3, intercellular colonization. Bars: 15 μm.

In order to test whether JA- or SA-related pathways contribute to controlling endophytic colonization of roots, we employed well-characterized rice mutants with altered hormone levels or signaling cascades. First, rice mutant *cpm2* (*coleoptile photomorphogenesis*) was tested, where the gene encoding allene oxide cyclase (AOC) in the JA synthesis pathway is disrupted, which results in a lack of JA production (Riemann et al., 2013). Colonization experiments were carried out in gnotobiotic culture systems with *A. olearius* BH72 or *Xoo,* respectively, and evaluated 14 days post-inoculation. The endophytic root colonization estimated by life cell counts was significantly increased (six-fold) in the jasmonate-deficient rice mutant in comparison to corresponding wild type cv. Nihonmasari (**Figure 4C**). Physiologically successful colonization was assessed by a reporter strain of *A. olearius* carrying a transcriptional fusion between the nitrogenase gene *nifH* and the ß-glucuronidase gene (Egener et al., 1999). Patterns of expression of nitrogen fixation genes were similar, with root tips and emergence points of lateral roots as main colonization and activity sites (**Figure 4C.1**), as well as intracellular (**Figure 4C.2**) and intercellular (**Figure 4C.3**) colonization. However, as expected from colonization quantification above, *nif-*gene expressing bacteria were more frequent and denser in *cpm2*-roots. In contrast, no significant effect of the *cpm2* mutation on root colonization of the pathogen was detected (**Figure 4A**), as expected from expression profiling. This suggests that the JA pathway controls to some extent the density of internal colonization of roots by the endophyte, while the pathogen appeared to overcome this control.

SA is playing a main role in plant defense against biotrophic or hemibiotrophic pathogens in *Arabidopsis*, with NPR1 (NONEXPRESSOR OF PATHOGENESIS-RELATED GENES 1) acting as a central regulator of salicylic-acid (SA)-mediated defense signaling. OsNPR1 is the rice ortholog of AtNPR1. It has been shown in previous studies that over-expression of *OsNPR1* conferred disease resistance to bacterial blight, but also enhanced herbivore susceptibility in transgenic plants (Yuan et al., 2007; Sugano et al., 2010). To test the impact of SA signaling on root colonization, two RNA interference (RNAi) knockdown mutants, *OsNPR1*-kd transgenic lines #1 and #7 (Sugano et al., 2010), were used. To verify the down-regulation of *OsNPR1*-expression in roots, transcript levels were quantified by RT-qPCR in our experimental system with and without inoculation of endophyte. In both lines, transcript levels were reduced as expected, even upon bacterial colonization (**Figure S6**). Endophytic colonization levels were not affected in the transgenic lines (**Figure 4A**), nor were *nifH* gene expression patterns altered (**Figure 4D**). Surprisingly, root colonization by the pathogen *Xoo* was decreased in knockdown lines, albeit only in line #7 at statistically significant levels (**Figure 4B**). In contrast, in rice leaves, RNAi lines showed enhanced disease susceptibility to *X. oryzae* (Yuan et al., 2007).

This highlights differences in defense responses in roots and shoots of rice. Also, in *Arabidopsis thaliana* and *Brassica* spp., the antagonistic interactions of the hormones JA and SA as well as their regulatory effects on defense genes was reported to differ between aerial and below-ground organs (Chuberre et al., 2018). For example, in rice, marker genes for defense responses *PR-1* and *PR-10* are transiently expressed during the early stages of root infection, while in leaves they continue to be transcribed during later stages of infection (Marcel et al., 2010). Concordantly, there were considerable differences in SA- and JA-related DEGs induced by *Xoo* according to our root data and published leaf data (RiceXpro, http://ricexpro.dna.affrc.go.jp/) (**Table S6**).

### Putative Metabolic Responses Affected by Endophyte and Pathogen

*X. oryzae* injects transcription activator-like (TAL) effector proteins into plant host cells to modulate gene expression and thereby the plant response. Among the induced targets are sugars transporter (SWEET) genes, for example *OsSWEET11* and *OsSWEET14* in leaves (Strom et al., 2001; Baker et al., 2003; Wilkins et al., 2015), which may lead to increase of the sugar levels in the apoplast serving as carbon source for the pathogen. Partially these mechanisms appear to occur also in roots: *OsSWEET11* and potential phosphate transporter encoding *Os06g29790,* which are only moderately induced in leaves (~10/2 fold) (Cernadas et al., 2014), were strongly induced in roots (70 fold, **Table S3**). Also, *OsSWEET6a/6b* which has not been reported to be affected in expression by *Xoo* in leaves, was upregulated by *Xoo* infection in roots. Interestingly, *Azoarcus* colonization resulted in down-regulation of two other *OsSWEET* genes, *OsSWEET15* (16.6-fold) and *OsSWEET1b* (1.9-fold). *OsSWEET15* was recently found to be able to support the *Xoo* virulence (Streubel et al., 2013). As *Azoarcus* does not grow on any carbohydrates (Reinhold-Hurek et al., 1993b) and would thus not profit from apoplastic sugars, we speculate that the endophyte could counteract carbohydrate supply to the pathogen.

Several other DEGs were also involved in the carbon metabolism (**Figure S7A**, **Table S8**). *Xoo* led to a strong induction of fermentative metabolism, indicated by a strong up-regulation of genes coding for PEP carboxykinase, lactate dehydrogenase ADH, and two pyruvate decarboxlyases. Also, the endophyte colonization induced alcohol dehydrogenase genes and decreased aldehyde dehydrogenase expression. This correlates well with the carbon sources preferences of *A. olearius* BH72: while malate is the preferred carbon source, ethanol is also readily metabolized, (Reinhold-Hurek et al., 1993a; Reinhold-Hurek et al., 1993b; Krause et al., 2011), especially during rice root colonization (Krause et al., 2011).

Among DEGs related to nitrogen metabolism, ammonium assimilation (glutamine synthetase, *OsGS2*), and aminotransferases expression was slightly decreased by the nitrogen-fixing endophyte, suggesting that at this stage, ammonium from nitrogen fixation might not be transferred. Interestingly, we have detected up-regulation of four genes encoding members of family of low affinity nitrate transporters/large peptide transporters (NTR1/PTR). While this family of transporters has 53 homologues in rice which exhibit various functions, it has been suggested (based on similarity to well-characterized members of this family in *Arabidopsis*) that *Azoarcus*-responsive genes *OsNRT1.1C* (5.2-fold up-regulated) and *OsNRT1.2* (1.6-fold up-regulated) could encode actual nitrate transporters (Plett et al., 2010). Moreover, the expression of seven uncharacterized amino-acid transporters was also induced by *Azoarcus* (**Table S7**, **Figure S7**).

### Comparison of Root Transcriptomic Responses to Other Microbes

In order to identify DEGs which might be specifically related to signal transduction in *Azoarcus-*rice interaction in contrast to pathogenic interactions, data for *Xoo*-rice leaf infection and *Magnaporthe oryzae*-rice root and leaf infection were included in the comparison (RiceXPro, ricexpro.dna.affrc.go.jp/). Only five DEGs were detected exclusively in the *Azoarcus*-rice root interaction: up-regulated DEGs coding for a LRR_RLK protein (*Os11g0208900*), *OsWAK103* (*Os10g0151100*), *OsERF86* (*Os07g0410700*), *OsCDPK25* (*Os11g0136600*), and a down-regulated DEG coding for an EF hand domain-containing protein (*Os09g0483300*). Leucine-rich-repeat receptor-like kinases like Os11g0208900 are typically involved in perception of MAMPs/PAMPs. Whether any of these candidate proteins is involved in specific *Azoarcus* or endophyte perception and signal transduction will have to be tested in further experiments. As a first step the data presented here were compared with rice transcriptome results published for *Azospirillum* spp. (Drogue et al., 2014). They are root-associated diazotrophs that are well-known as phytostimulators (Okon and Labandera-Gonzalez, 1994; Cerezini et al., 2016), and plant growth promotion effects are mainly attributed to production of the phytohormone IAA and the modulation of the plant phytohormonal balance rather than nitrogen fixation (Steenhoudt and Vanderleyden, 2000; Somers et al., 2005). *Azospirillum lipoferum* 4B is an efficient rhizoplane colonizer (Drogue et al., 2014), while *Azospirillum* sp. B510 originates from surface-sterilized rice roots and is an endophyte of rice (Yasuda et al., 2009; Kaneko et al., 2010). Interestingly, there were no overlaps in genes related to signal perception and transduction modulated by both these strains and *Azoarcus.* However, comparison of DEGs in response to only endophytic strains BH72 and B510 revealed commonalities (**Table S9**). Both endophytes induced jasmonate-dependent responses, repressed DEGs for cell wall degradation, and downregulated genes related to photosynthesis. The latter is likely to be related to the jasmonate pathway, as both, nuclear and plastid photosynthetic genes, are repressed under the control of JA (Reinbothe et al., 2009). Although in contrast to *Azospirillum, A. olearius* is not known to produce IAA (Krause et al., 2006), both strains repressed *OsIAA9, Os02g0805100* encoding an auxin responsive protein. The otherwise strongly strain-specific and cultivar-specific rice responses (Drogue et al., 2014) indicate consequences of different epiphytic and endophytic lifestyles, but also that individual genotypic variations of the host plants may be important driving forces in the cooperation with beneficial bacteria.

## Concluding Remarks

Plants are encountering a vast diversity of microorganisms in roots in comparison to the foliar region, including beneficial bacteria and fungi as well as both prokaryotic and eukaryotic pathogens (Mendes et al., 2013). The high density of bacterial colonization calls for a reduced sensitivity of roots toward microbial molecules and of defense responses, which may account for deviating hormonal responses in below- and above-ground tissues. Furthermore, different panels of host gene transcription are induced during root colonization by beneﬁcial or detrimental microbes (Plett and Martin, 2018). For fungi having a pathogenic (*Magnaporthe grisea* and *Fusarium moniliforme*) or a symbiotic lifestyle (arbuscular mycorrhiza fungus *Rhizophagus irregularis*), an overlap of only 13% of DEGs was found in rice roots (Guimil et al., 2005). In case of the bacterial endophyte *A. olearius* BH72 compared to another *Proteobacterium, X. oryzae*, the overlap was even smaller (8% of the endophyte-, 6% of the pathogen-modulated DEGs), which demonstrates strong deviation of the lifestyle in a more “loose” beneficial interaction.

As observed for symbiotic interactions (Duplessis et al., 2005), defense reactions are provoked during the early phases of contact. A strong time-dependent modulation of expression of *OsJAR1* gene was detected in experiments presented here, which raises the question: at which stages and how do endophytes attenuate defense? To elucidate pathways which could perceive and transduce signals specific for endophytic colonization, putative candidate genes should to be verified in transcriptomes at different time points and genotype combinations. How mutualistic microbes modulate defense responses—through effector proteins, siRNAs, or other molecules, is still not clear.

One of the key findings by using rice mutants with altered hormonal responses is that JA signaling is involved in controlling the *Azoarcus* endophyte density in roots and thus contributes to shaping the root microbiome. Colonization assays using rice mutants deficient in jasmonate synthesis (*cpm2*) or exhibiting reduced salicylic acid-mediated defense responses (*NPR1-kd*) suggested that endophytic colonization is controlled through mechanisms which involve JA-production and signaling and are SA-independent. *Xoo* colonization did not appear to be subject to these control mechanisms. Previous studies using plant mutants of *Arabidopsis* demonstrated that salicylic acid is the major hormonal pathway that modulates the community composition at roots (Lebeis et al., 2015). However, external addition of methyl jasmonate also affected the community structure of *Arabidopsis* rhizosphere soil (Carvalhais et al., 2013), and wheat roots (Liu et al., 2017). Unfortunately, these studies did not address quantitation of endophytic colonization. Furthermore, the endophytic compartment was not well differentiated because ultrasonication was used to remove surface bacteria, which is not very efficient in soil-based settings (Reinhold-Hurek et al., 2015). According to the presented data for *Azoarcus*, the JA pathway appears to restrict the internal root colonization, probably below a limit which may become harmful to the plant. Deeper knowledge of the molecular mechanisms, especially time-resolved responses, identification of endophyte-specific perception proteins, and bacterial signals involved, may help to modulate the endophyte microbiome for improved biotechnological applications.

## Data Availability Statement

The datasets generated for this study can be found in the GEO (GSE136706 and GSE136707).

## Author Contributions

XC and BR-H designed the experiments. XC carried out most experiments. MM performed the timeline experiments. XC, MM, and BR-H wrote the manuscript.

## Funding

The work was supported by a grant of the Deutsche Forschungsgemeinschaft DFG to BR-H (Re756/18-1), and the University of Bremen (02/124/10).

## Conflict of Interest

The authors declare that the research was conducted in the absence of any commercial or financial relationships that could be construed as a potential conflict of interest.
