# Supplementary material for: Jasmonic Acid, Not Salicyclic Acid Restricts Endophytic Root Colonization of Rice
Source: Front Plant Sci. 2020 Jan 29;10:1758. doi: 10.3389/fpls.2019.01758 (PMC7000620; doi:10.3389/fpls.2019.01758)
Supplement: Supplementary file 1 [file DataSheet_1.pdf]

Supplementary Figures Chen *et al.*

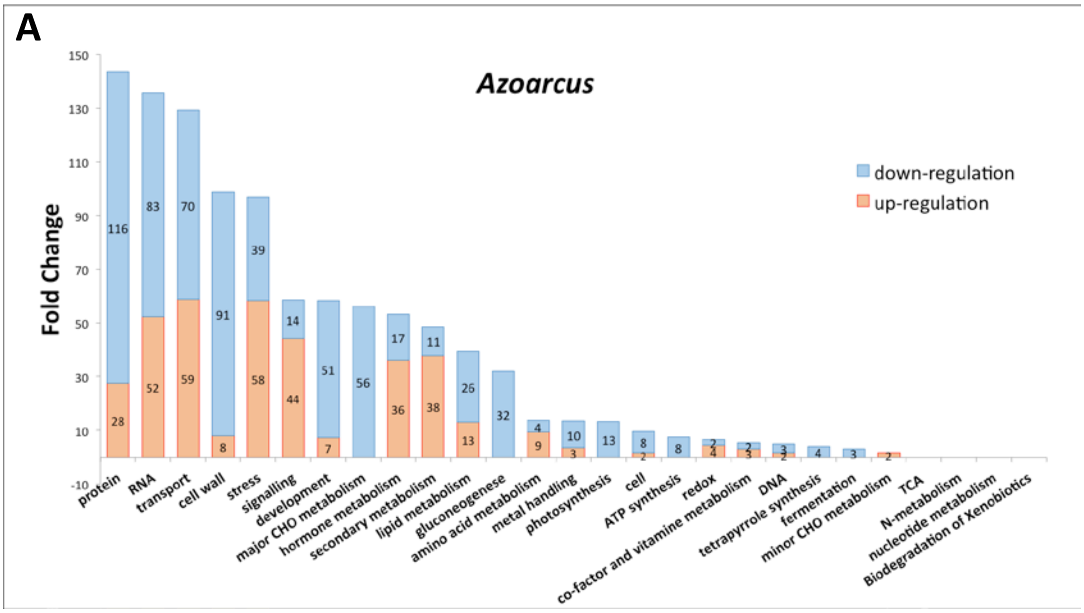

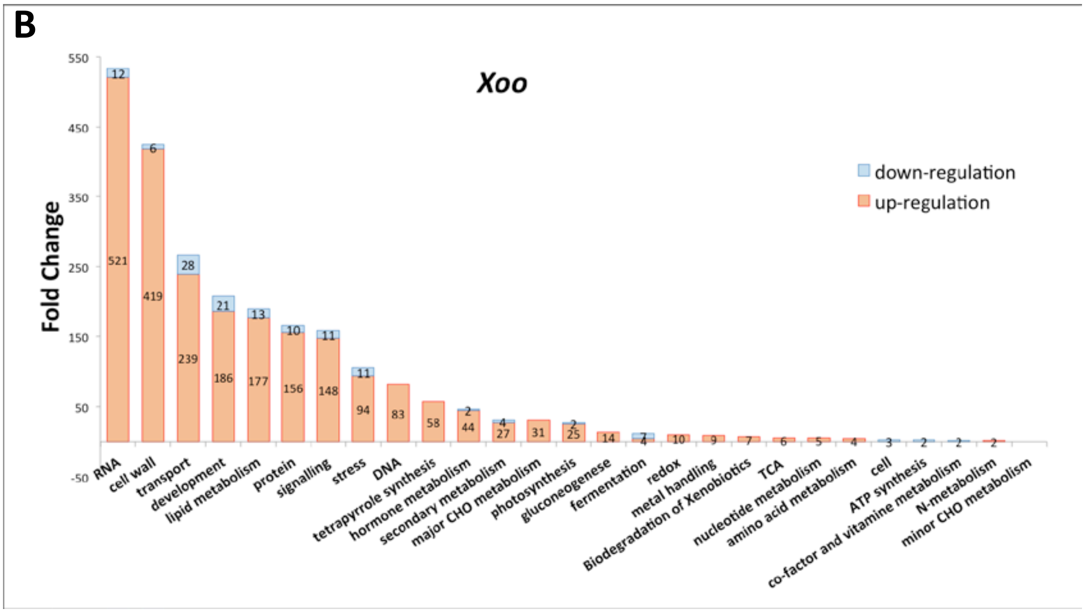

Figure S1. Sum of fold change of DEGs in rice roots assigned to Kyoto Encyclopedia of Genes and Genomes function (KEGG) categories. A. *Azoarcus* colonization, **B.** *Xoo* infection; vertical axis, sum of fold change of all the genes in the same functional category; horizontal axis, functional categories based on KEGG categories. 65% of genes differently expressed in *Azoarcus* colonization and 56% in *Xoo* were assigned into at least one functional category.

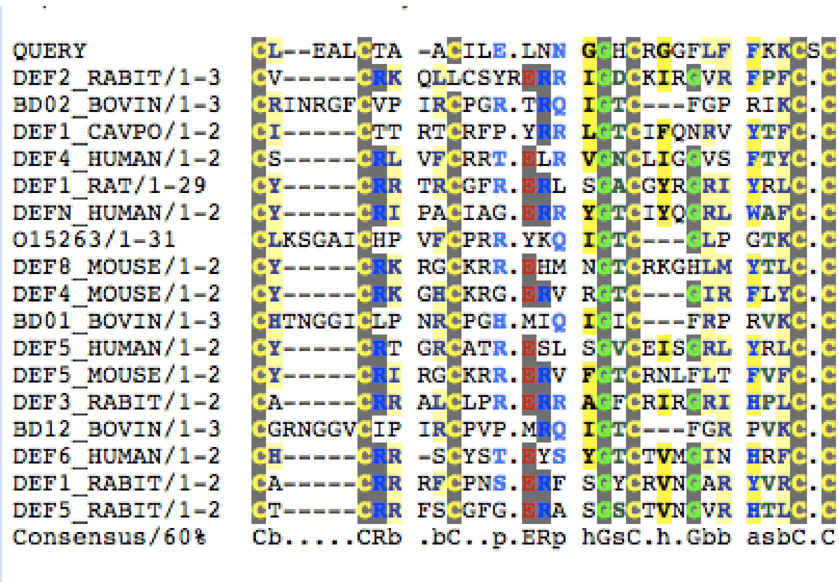

Figure S2. Alignment of Os04g0381500 protein sequence with other defensins. Protein sequences removed of signal peptides were used for alignment. Alignment was performed using the Simple Modular Architecture Research Tool (SMART, http://smart.embl-heidelberg.de/). Defensins are commonly consisting of 45-70 amino acids and possess four disulphide bonds, but NCRs usually contain just 30-55 amino acids and two to three disulphide bonds. A distinguishing character of NCRs from defensins is four or six conserved cysteine residues in a special pattern. The typical cysteine amino acids are highlighted in yellow color above brown background.

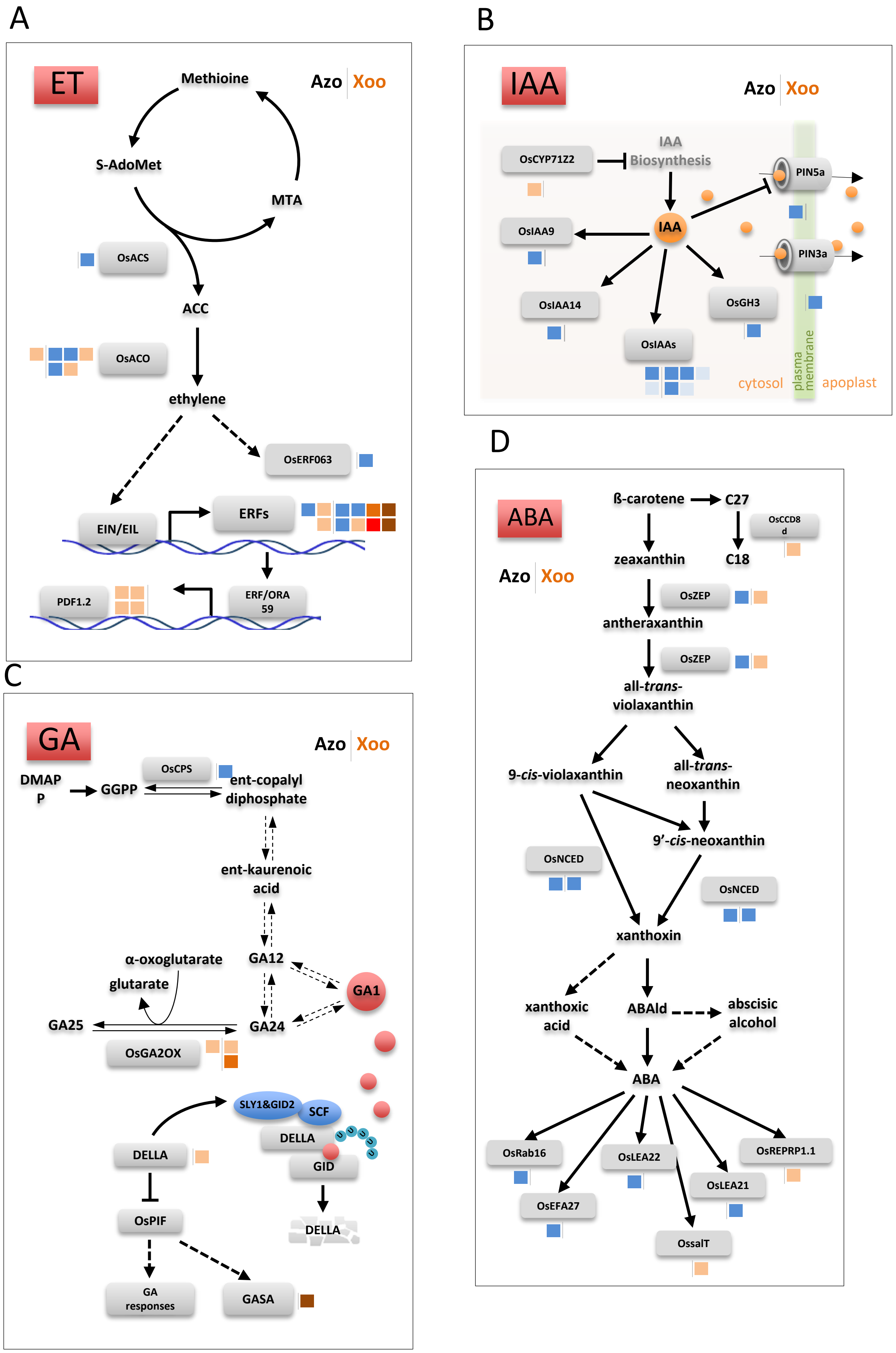
A
ET
Azo Xoo
Methioine
S-AdoMet
MTA
OsACS
ACC
OsACO
ethylene
OsERF063
EIN/EIL
ERFs
PDF1.2
ERF/ORA 59
B
IAA
Azo Xoo
OsCYP71Z2
IAA Biosynthesis
PIN5a
OsIAA9
IAA
PIN3a
OsIAA14
OsGH3
OsIAAs
cytosol
plasma membrane
apoplast
C
GA
Azo Xoo
OsCPS
DMAP P
GGPP
ent-copalyl diphosphate
ent-kaurenoic acid
α-oxoglutarate
glutarate
GA12
GA1
GA25
GA24
OsGA2OX
SLY1&GID2
SCF
DELLA
GID
DELLA
DELLA
OsPIF
GA responses
GASA
D
ABA
Azo Xoo
ß-carotene
C27
OsCCD8 d
zeaxanthin
C18
OsZEP
antheraxanthin
OsZEP
all-trans-violaxanthin
9-cis-violaxanthin
all-trans-neoxanthin
9'-cis-neoxanthin
OsNCED
OsNCED
xanthoxin
xanthoxic acid
ABAld
abscisic alcohol
ABA
OsRab16
OsLEA22
OsREPRP1.1
OsEFA27
OsLEA21
OssalT

E

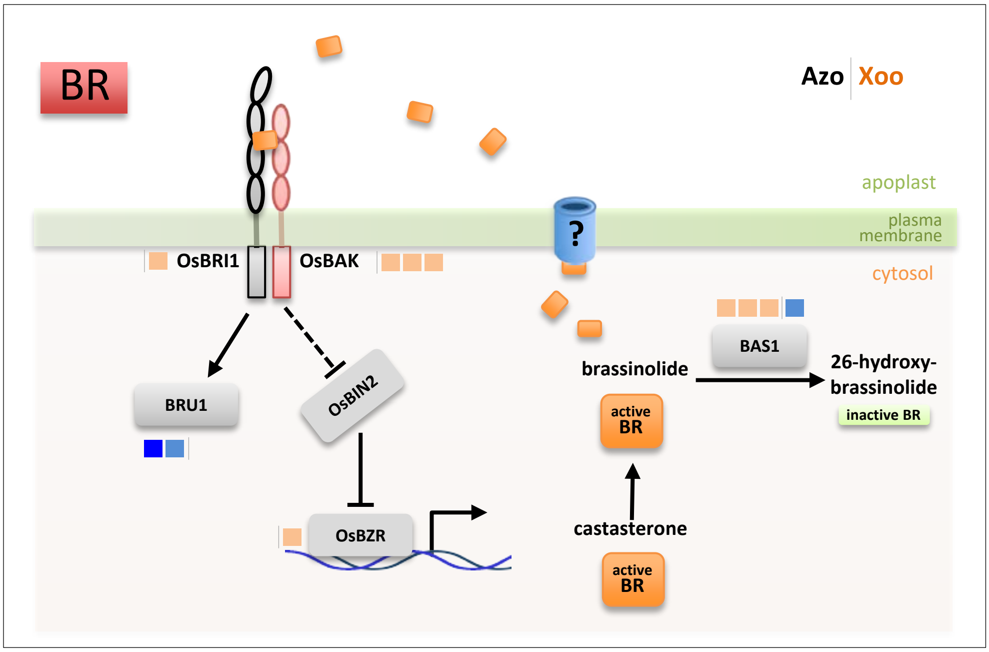
BR
Azo
Xoo
apoplast
plasma membrane
cytosol
OsBRI1
OsBAK
?
BRU1
OsBIN2
OsBZR
BAS1
brassinolide
26-hydroxy-brassinolide
inactive BR
active BR
castasterone
active BR

F

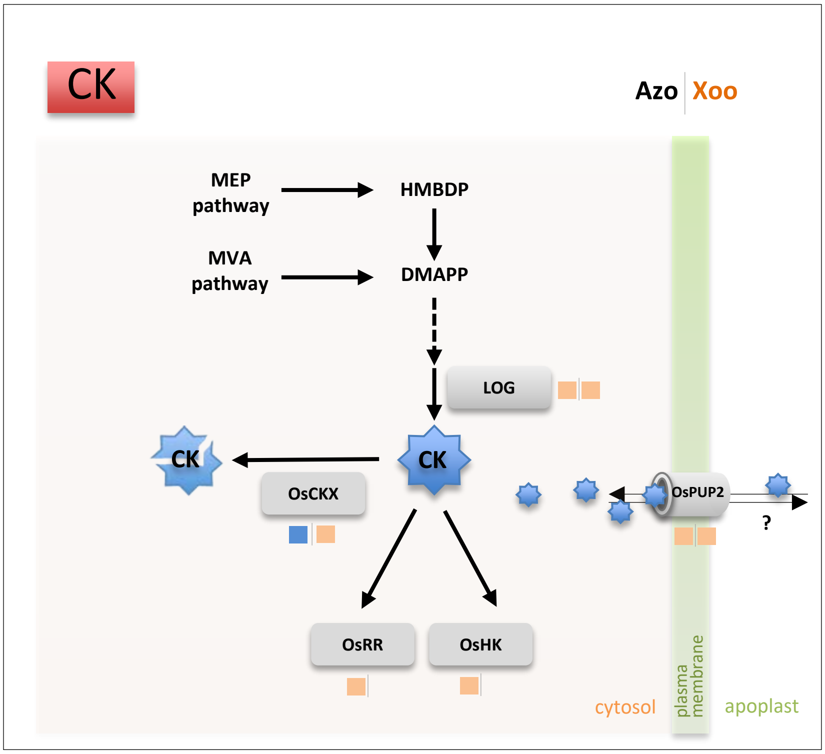
CK
Azo
Xoo
MEP pathway
HMBDP
MVA pathway
DMAPP
LOG
CK
CK
OsCKX
OsPUP2
?
OsRR
OsHK
cytosol
plasma membrane
apoplast

Figure S3. Regulation of genes related to hormone synthesis and downstream reactions. Except for SA and JA, seen **Figure 3** and **Figure S4.** Next to rice protein names small blocks representing Fold change of the DEG (right side of the vertical line, modulated by *Xoo*; left side, by *Azoarcus*) according to the color scale below. Dashed line: steps omitted, continuous line: direct reaction, arrow: reaction or induction, blunt end: inhibition. FC and annotation of DEGs from Table S5. Abbreviations: ABAld: abscisic aldehyde, ACO: 1-aminocyclo-propane-1-carboxylic acid (ACC) oxidase, ACS: aminocyclopropane-1-carboxylic acid (ACC) synthase, BAK: BRASSINOSTEROID INSENSITIVE 1 (BRI1)-associated receptor kinase, BIN: brassionsteroid insensitive, BR: brassinosteroid, BRCA: breast cancer, BRI: BR insensitive, BRU: BR-unregulated gene, CA: cinnamic acid, CCD: carotenoid cleavage dioxygenases, CKX: cytokinin oxidase/ dehydrogenase, CPS: copalyl diphosphate synthase, CRK: cysteine-rich receptor-like protein kinase, CYP: cytochrome P450, DELLA: Asp (D)-Glu (E)-Leu (L)-Leu (L)-Ala (A), DMAPP: dimethylallyl pyrophosphate, EFA: EF-hand, abscisic acid responsive 27-kDa protein, EIL: ethylene insensitive-3 (EIN3)-like, EIN: ethylene insensitive, ERF: ethylene response transcription factor, GA: gibberellic acid, GA2OX: GA 2-oxidase, GGPP: geranylgeranyl diphosphate, GID: GA insensitive dwarf, HMBDP: 1-hydroxy-2- methyl-2-(E)-butenyl 4-diphosphate diphosphate, IAA: indole-3-acetic acid, IC: isochorismate, LEA: late embryogenesis abundant protein, LOG: lonely guy like phosphoribohydrolase, MVA: mevalonic acid, NAC: no apical meristem (NAM), *Arabidopsis* transcription activation factor (ATAF) and cup-shaped cotyledon (CUC) transcription factor family, NCED: 9-cis-epoxycarotenoid dioxygenase, NPR: nonexpressor of pathogenesis-related genes, Os: *Oryza sativa*, PAL: phenylalanine ammonia lyase, Phe: phenylalanine, PIF: phytochrome interacting factor, PIN: passively transports IAA out, PR: pathogenesis related, PUP: purine permease, Rab: responsive to abscisic acid, REPRP: repetitive proline-rich protein, SCF: Skp, Cullin, F-box containing complex, SLY: sleepy, SSI: suppressor of salicylic acid insensitivity.

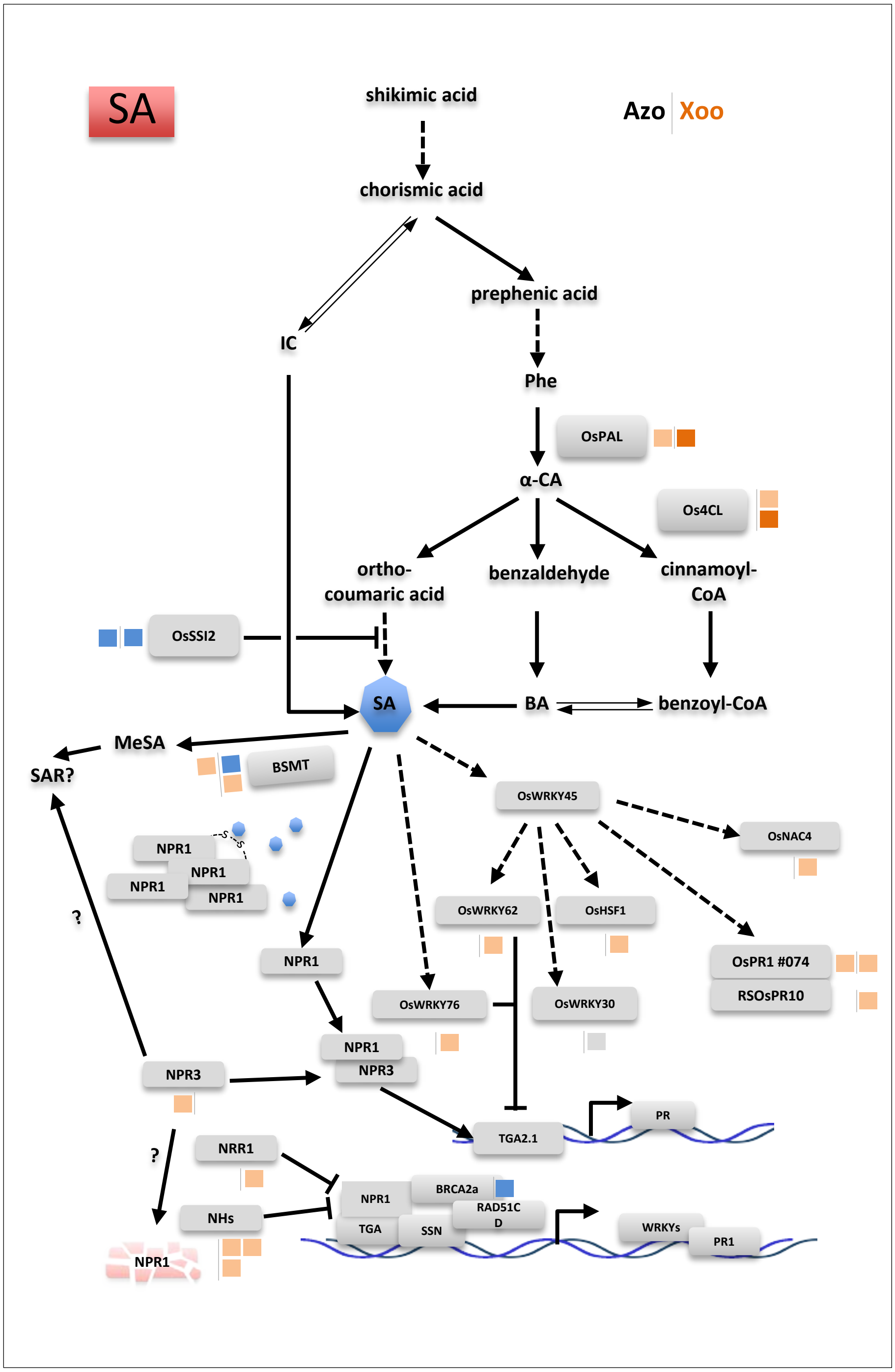

Figure S4. Regulation of genes related to SA synthesis and downstream reactions. Next to rice protein names small blocks representing Fold change of the DEG (right side of the vertical line, modulated by *Xoo*; left side, by *Azoarcus*) according to the color scale below. Dashed line: steps omitted, continuous line: direct reaction, arrow: reaction or induction, blunt end: inhibition. FC and annotation of DEGs from Table S5. Abbreviations: 4CL: 4-coumarate: coenzyme A ligase, BRCA2: BRCA2 repeat containing protein, BSMT: benzoic acid/salicylic acid methyltransferase, CoA: coenzyme A, NRR: negative regulator of resistance, Os: *Oryza sativa*, RS: root specific, SSN: suppressor of sni1, TGA: TGACGTCA cis-element-binding protein, WRKY: amino-acid sequence WRKYGQK.

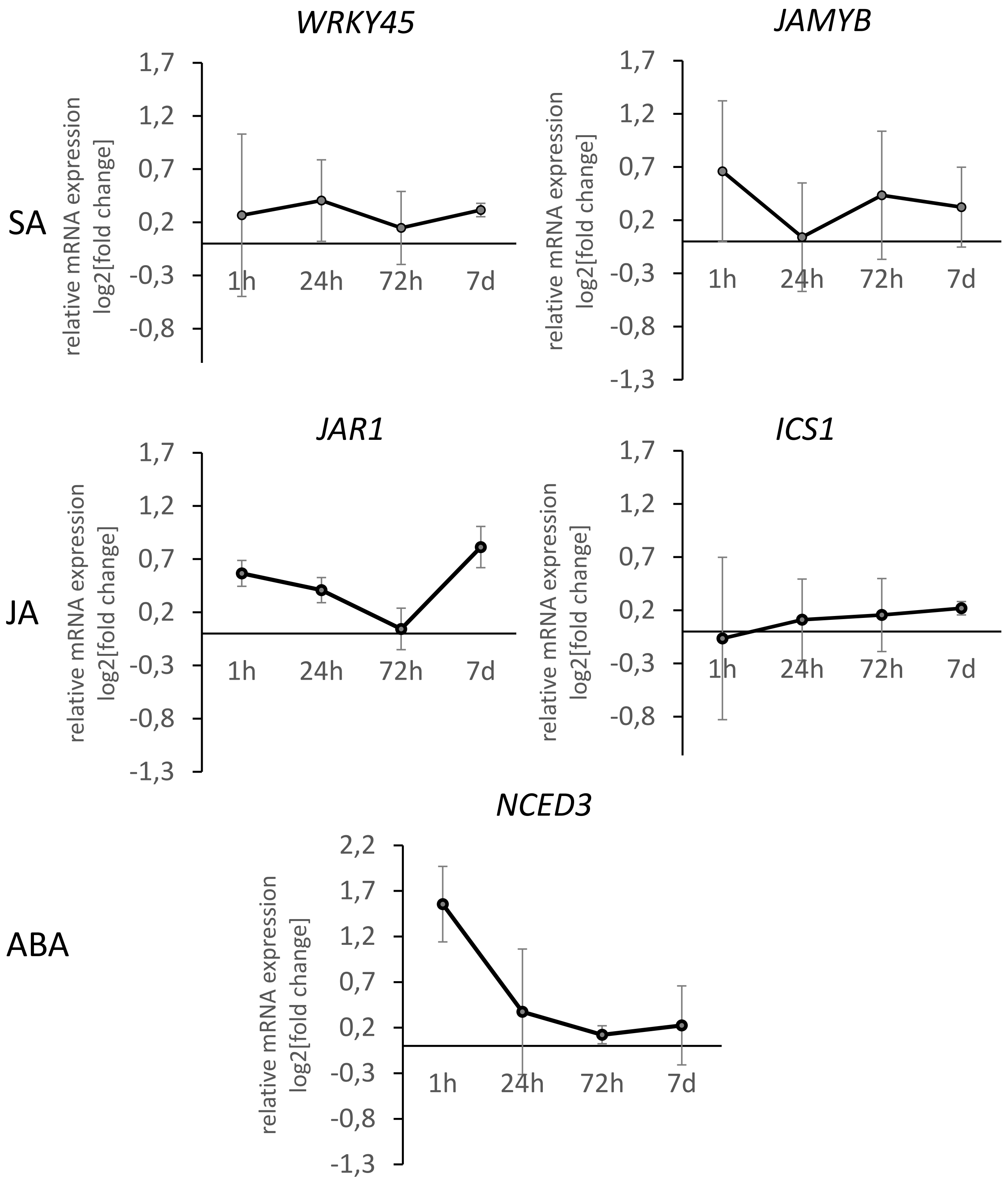

Figure S5. Transcript level of genes involved in hormone biosynthesis and downstream reaction in a timeline *Azoarcus* colonization experiment. Roots harvested for RNA extracted 1 h, 24 h, 72 h and 7 d post inoculation, with uninoculated controls. Transcript levels normalized to *UBQ5* according to Jain *et al*. 2006). Results of three independent biological replicates.

Jain, M., Nijhawan, A., Tyagi, A.K., and Khurana, J.P. (2006). Validation of housekeeping genes as internal control for studying gene expression in rice by quantitative real-time PCR. *Biochem. Biophys. Res. Comm.* 345**,** 646-651. doi: S0006-291X(06)00968-5 10.1016/j.bbrc.2006.04.140.

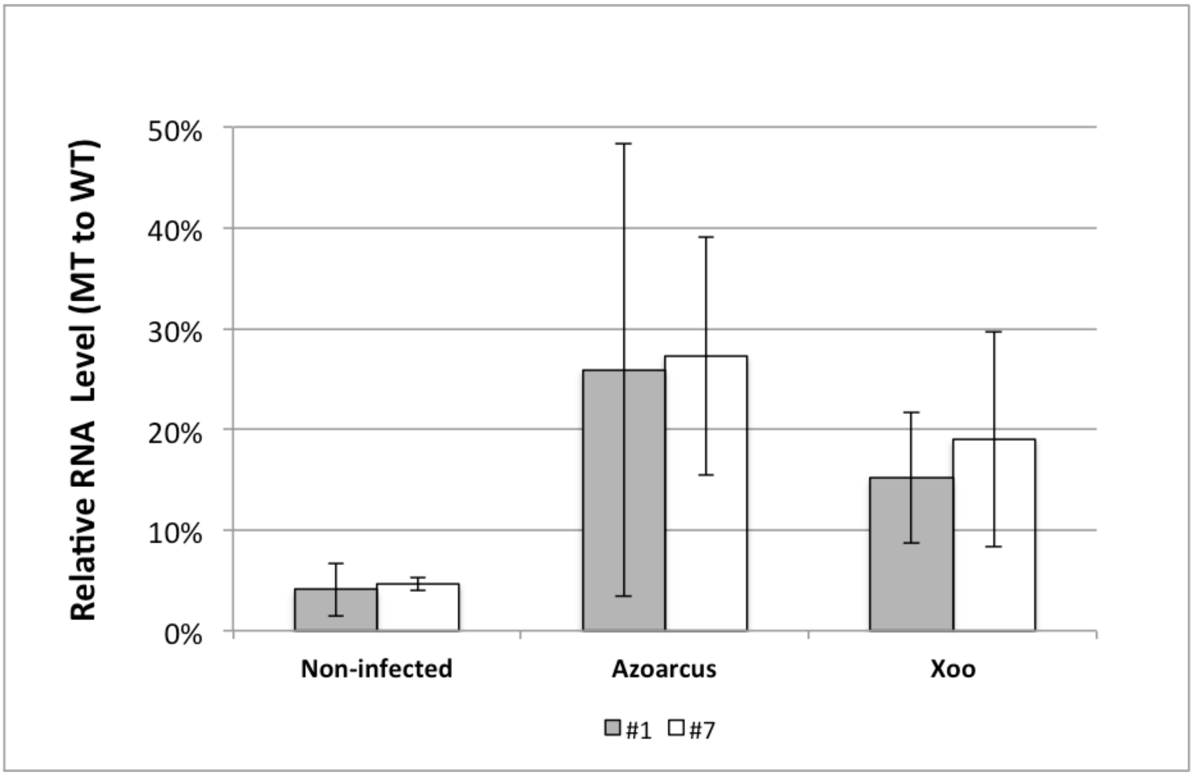

Figure S6. Control of *OsNPR1* expression in roots of rice wild type cv. Nipponbare and knockdown lines *npr1#1* and *npr1#7.* Plants grown under sterile or gnotobiotic conditions inoculated with *A. olearius* BH72 or *X. oryzae* PXO99, respectively. RNA extracted from roots 14 d post inoculation, and subjected to RT-qPCR analysis for *OsNPR1* expression normalized to ubiquitin gene expression. Relative levels of expression in mutant lines in comparison to wild type.

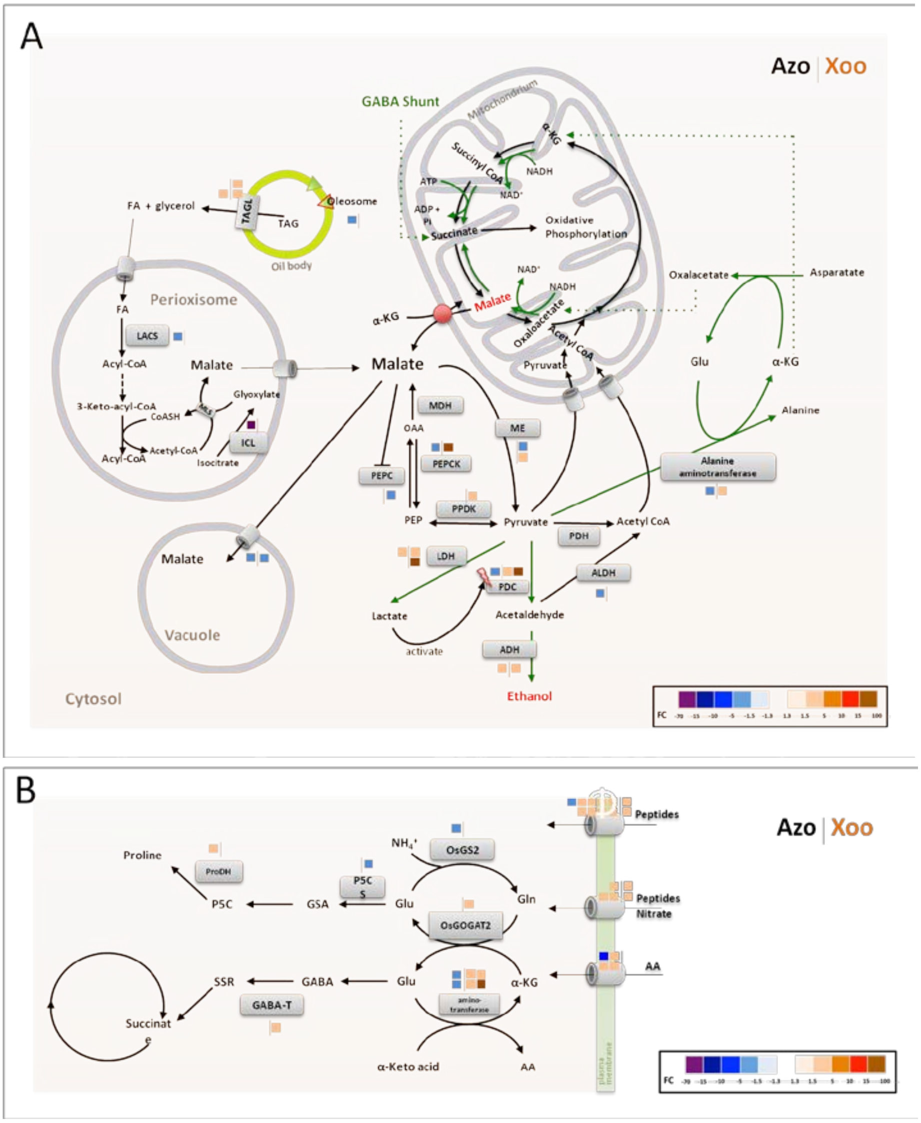

Figure S7. Carbon (A) and nitrogen (B) metabolism in rice cells affected by *Azoarcus*- and *Xoo.* Gene expression changes upon inoculation with *A. olearius* BH72 or *Xoo*, respectively, according to color code. Abbreviations: AA: amino acid, ADH: alcohol dehydrogenase, ADP: adenosine diphosphate, ALDH: aldehyde dehydrogenase, ATP: adenosine triphosphate, CoA: coenzyme A, FA: fatty acid, GABA: g-aminobutyric acid, GABA-T: GABA transaminase, Gln: glutamine, Glu: glutamate, GOGAT: Glutamine oxoglutarate aminotransferase, GS: glutamine synthetase, GSA: galactinol synthase A, ICL: Isocitrate lyase, KG: ketoglutarate, LACS: Long-chain acyl-CoA synthetase, LDH: lactate dehydrogenase, MDH: malate dehydrogenase, ME: malic enzyme, MLS: malate synthase, NAD: nicotinamide adenine dinucleotide, OAA: oxaloacetate, P5CS: Δ^1-pyrroline-5-carboxylate synthase, PDC: pyruvate dehydrogenase complex, PDH: pyruvate dehydrogenase, PEPC: phosphoenolpyruvate carboxylase, PEPCK: Phosphoenolpyruvate carboxykinase, PPCK: PEPC kinase, PPDK: pyruvate phosphate dikinase, ProDH: prolin dehydrogenase, SSR: succinate reductase, TAG: triacylglycerol, TAGL: TAG lipase.
