# Supplementary material for: Jasmonic Acid, Not Salicyclic Acid Restricts Endophytic Root Colonization of Rice
Source: Front Plant Sci. 2020 Jan 29;10:1758. doi: 10.3389/fpls.2019.01758 (PMC7000620; doi:10.3389/fpls.2019.01758)
Supplement: Supplementary file 2 [file DataSheet_2.pdf]

Table S1. Primer sequences applied in real-time PCR.

Locus Number	Description	Primer-Forward	Primer-Reverse	
**Os09g0361500**	OsICS1	TGTCCCCACAAAGGCATCCTGG	TGGCCCTCAACCTTTAAACATGCC	
**Os05g0322900**	OsWRKY45	GAATCATGGATGGACACGGG	AGACCCCCAGCTCATAATCA	
**Os05t0586200**	OsJAR1	GATCCCAGCACTGTGGCCAA	CCGATGCGCCATAGTCAGCA	(Chen *et al* . 2015)
**Os11g0684000**	OsJAmyb	GGACCTCACCCTCATCAATTA	TGCTATCTTGGACCATCGGTT	(Chen *et al* . 2015)
**Os04g0578000**	OsACS2	TGCGCCTTACTACGTCGACTACAT	ACGCACTAACGCACGTCTCTACAA	
**Os08g0203400**	SHR5	ATCTCTTTGAGTGGGCTTGGA	CTGCTGAGCTTAGGGGTAGT	
**Os03g0645900**	OsNCED3	CTACATCCTGTCCTTCGTCCAC	AGAAACGTGGAGGTGTTCGAT	
**Os03g0285800**	OsMAPK5	GGGATCGTCTGCTCCGTGATGA	ATGTTCTCGTGGTCGAGGTGCC	
**Os02g0587800**	Virulence factor, pectin lyase fold family protein	CGATGACGACCCTATTGGCTA	CAGTCCGCTCTATCGCTCTT	
**Os03g0667100**	OsNPR3	CGCTCCTCGTTTCAACAGCTC	TTGGTGCATTGACAGAGTGCCA	
**Os05g0368000**	RH1	CTCGTTGGAATTGGTCCTTCGG	CTGGCACATGGATTGCGTGA	
**Os06g0317200**	Similar to glycine-rich cell wall structural protein	AGGCTAGCAAGCTCGACTGA	TCCCCTCAGATTCACTGGCTTC	
**Os06g0592500**	responsive transcriptional coactivator	TTAGGCGTTGATCCGTTTGTGAAT	GCCATCATCACTTCACATGCGTC	
**Os06g0695300**	prx92	GACTCCGTCTTCCTGAGTGGA	GAGGCAGGTTGACGCTGTTC	
**Os08g0535200**	Xa13, OsSWEET11	CCAGTAGCAATGGCAGGAGGT	AACGTCGCCACTGGTGCAA	
**Os09g0483300**	calcium-binding EF hand family protein	GGAGCAGCTGCCTAAACTGAAC	ACACCGTGTATCCTTCCGTGTAT	
**Os11g0242800**	ASCAB9-A, PSII CP26, PSII Lhcb5	CCGGGAAGAAGAAGGGAGGA	TCTCAGCCACAAATCCAAACACT	
**Os01g0194300**	OsNPR1	GTTCATGGCGCAGGTCCTCT	GCCTCGCAGCAATGTGAAGA	

Chen, X., Miché, L., Sachs, S., Wang, Q., Buschart, A., Yang, H., et al. (2015). Rice responds to endophytic colonization which is independent of the common symbiotic signaling pathway. *New Phytol.* 208**,** 531–543.
